# Correction to: Draft genome sequences of Hirudo medicinalis and salivary transcriptome of three closely related medicinal leeches

**DOI:** 10.1186/s12864-020-06897-0

**Published:** 2020-07-22

**Authors:** Vladislav V. Babenko, Oleg V. Podgorny, Valentin A. Manuvera, Artem S. Kasianov, Alexander I. Manolov, Ekaterina N. Grafskaia, Dmitriy A. Shirokov, Alexey S. Kurdyumov, Dmitriy V. Vinogradov, Anastasia S. Nikitina, Sergey I. Kovalchuk, Nickolay A. Anikanov, Ivan O. Butenko, Olga V. Pobeguts, Daria S. Matyushkina, Daria V. Rakitina, Elena S. Kostryukova, Victor G. Zgoda, Isolda P. Baskova, Vladimir M. Trukhan, Mikhail S. Gelfand, Vadim M. Govorun, Helgi B. Schiöth, Vassili N. Lazarev

**Affiliations:** 1 Federal Research and Clinical Centre of Physical-Chemical Medicine of Federal Medical Biological Agency, 1a Malaya Pirogovskaya Str, Moscow, 119435 Russia; 2 Koltzov Institute of Developmental Biology, Russian Academy of Sciences, 26 Vavilov str, Moscow, 119334 Russia; 3 Moscow Institute of Physics and Technology, 9 Institutskiy per., Dolgoprudny, Moscow Region 141700 Russia; 4 Vavilov Institute of General Genetics, Russian Academy of Sciences, 3 Gubkina str, Moscow, 119991 Russia; 5 A.A. Kharkevich Institute for Information Transmission Problems, Russian Academy of Sciences, 19 Bol’shoi Karetnyi per, Moscow, 127051 Russia; 6 Skolkovo Institute of Science and Technology, 3 Nobelya Ulitsa str, Moscow, 121205 Russia; 7 Shemyakin-Ovchinnikov Institute of Bioorganic Chemistry, Russian Academy of Sciences, 16/10 Miklukho-Maklaya str, Moscow, 117997 Russia; 8 V.N. Orekhovich Research Institute of Biomedical Chemistry, Russian Academy of Medical Sciences, 10 Pogodinskaja str, Moscow, 119832 Russia; 9 Faculty of Biology, Lomonosov Moscow State University, 1-12 Leninskie Gory, Moscow, 119991 Russia; 10 I.M. Sechenov First Moscow State Medical University of the Ministry of Healthcare of the Russian Federation (Sechenovskiy University), Trubetskaya str., 8-2, Moscow, 119991 Russia; 11 Faculty of Computer Science, National Research University Higher School of Economics, 20 Myasnitskaya str, Moscow, 101000 Russia; 12 Faculty of Bioengineering and Bioinformatics, Lomonosov Moscow State University, 1-73 Leninskie Gory, Moscow, 119991 Russia; 13 Functional Pharmacology, Department of Neuroscience, Uppsala University, Husargatan 3, 75124 Uppsala, Sweden

**Correction to: BMC Genomics (2020) 21:331**

**https://doi.org/10.1186/s12864-020-6748-0**

Following the publication of the original article [1], it was noted that due to a typesetting error that Fig. 1 was erroneously published as a duplicate of Fig. 5.

**Fig. 1:**
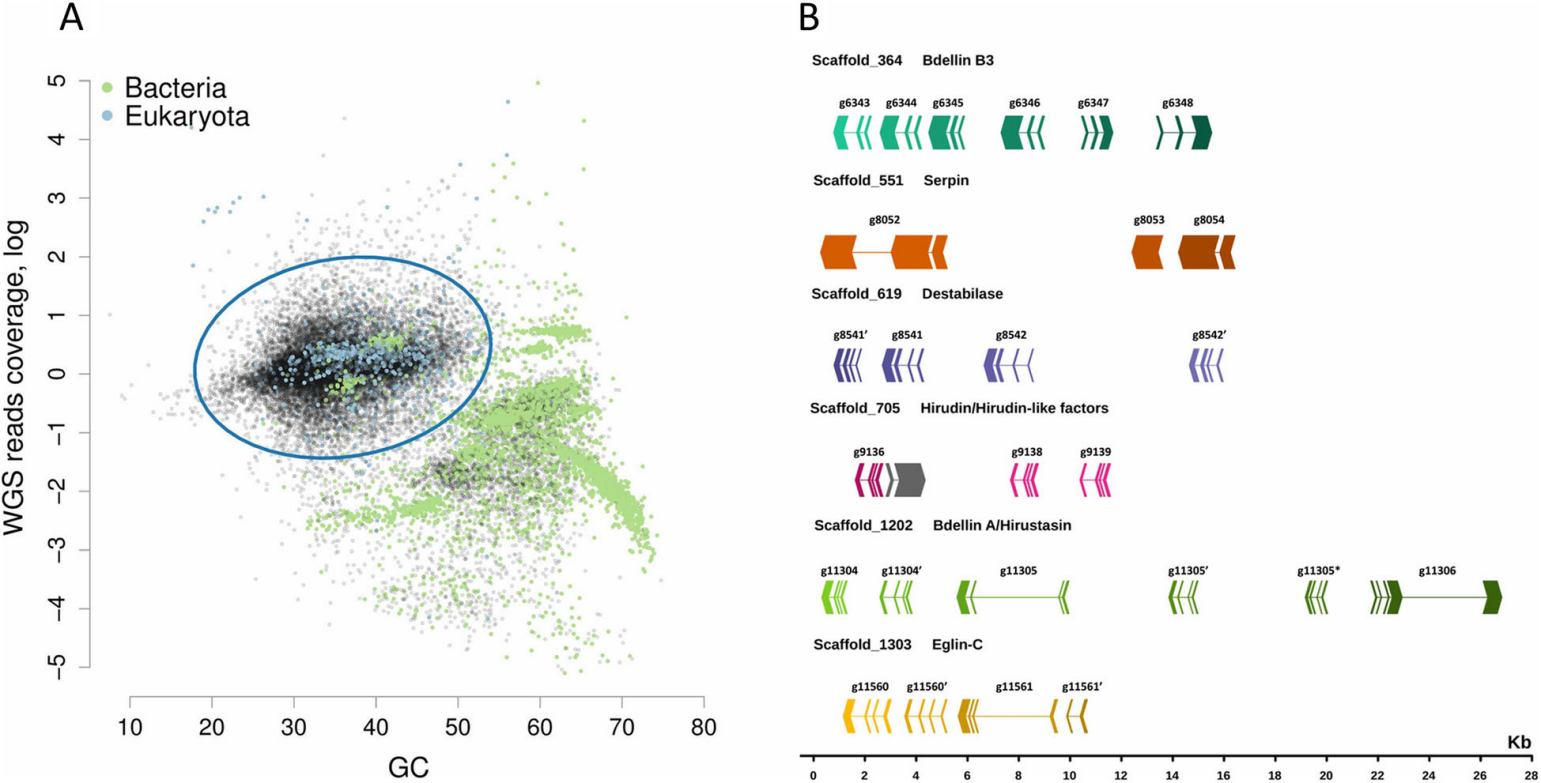
The *H. medicinalis* genome binning. **a** 2D-plot showing the contig distribution in coordinates of GC content and coverage by a combination of reads obtained by Ion Proton and Illumina. Contigs are indicated by dots, and the taxonomic affiliation of contigs at the domain level is encoded by colour (green – Bacteria, blue – Eukarya, black – no assignment). The taxonomic affiliation was determined by direct BlastN (megablast) search against the National Center for Biotechnology Information (NCBI) nt database. The 3D plot showing the contig distribution in coordinates of GC content, read coverage (Proton and Illumina), and host cDNA read coverage is presented in Supplementary Data 2. **b***H. medicinalis* genome contains clusters of blood meal-related genes. The graph shows the exon-intron structure of genes and arrangement of gene clusters in scaffolds on a general scale. The exon arrows indicate the direction of transcription (gray - unknown gene)

The correct Fig. 1 has been included in this correction, and the original article has been corrected.

The publisher apologizes to the authors and readers for the inconvenience.
