# Supplementary material for: Draft genome sequences of Hirudo medicinalis and salivary transcriptome of three closely related medicinal leeches
Source: BMC Genomics. 2020 Apr 29;21:331. doi: 10.1186/s12864-020-6748-0 (PMC7191736; doi:10.1186/s12864-020-6748-0)
Supplement: Supplementary file 1 — Additional file 1. Supplementary tables, figures, results and methods. [file 12864_2020_6748_MOESM1_ESM.pdf]

Supplementary Information

Draft genome sequences of *Hirudo medicinalis* and salivary transcriptome of three closely related medicinal leeches.

Vladislav V. Babenko, Oleg V. Podgorny, Valentin A. Manuvera, Artem S. Kasianov, Alexander I. Manolov, Ekaterina N. Grafskaia, Dmitriy A. Shirokov, Alexey S. Kurdyumov, Dmitriy V. Vinogradov, Anastasia S. Nikitina, Sergey I. Kovalchuk, Nickolay A. Anikanov, Ivan O. Butenko, Olga V. Pobeguts, Daria S. Matyushkina, Daria V. Rakitina, Elena S. Kostryukova, Victor G. Zgoda, Isolda P. Baskova, Vladimir M. Trukhan, Mikhail S. Gelfand, Vadim M. Govorun, Helgi B. Schiöth, Vassili N. Lazarev

Supplementary Figures

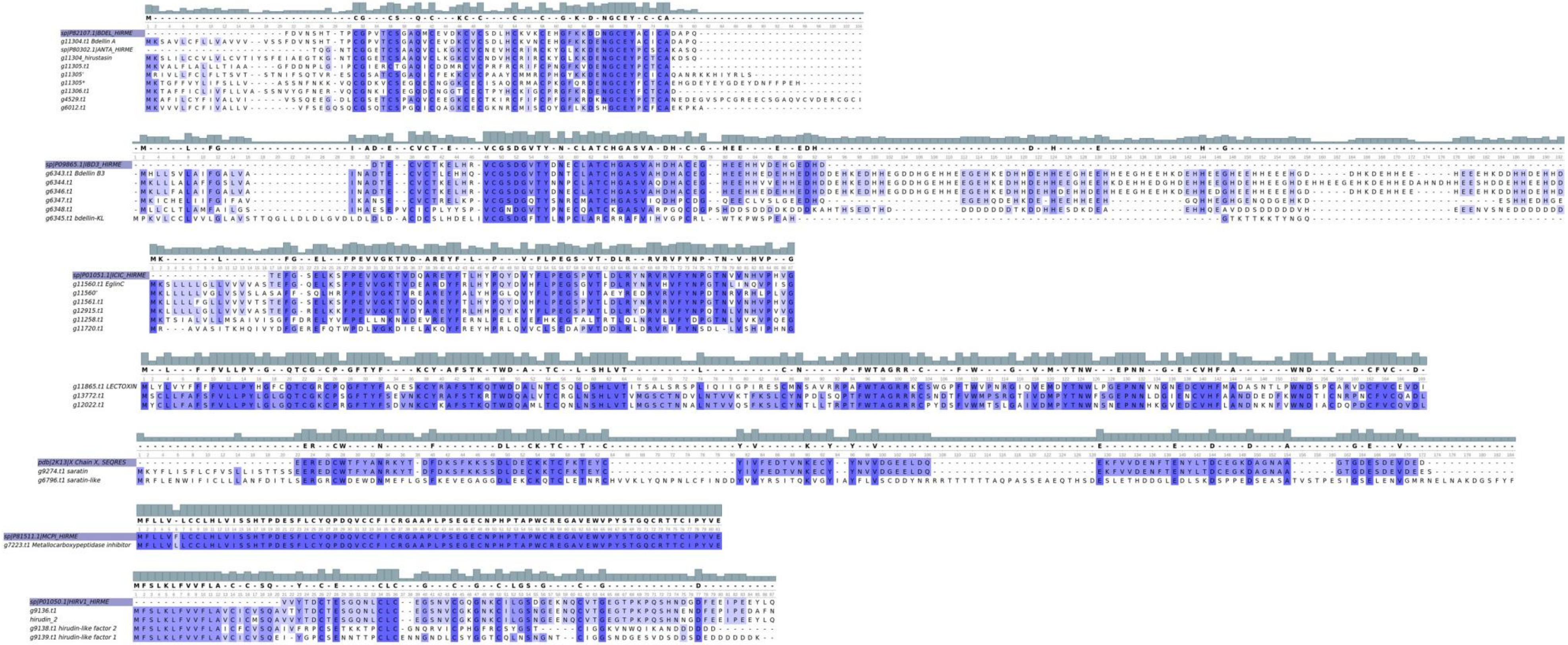

Supplementary Fig. 1. The protein alignment (with percentage Identity coloring scheme) of new homologs of genes encoding known anticoagulants or blood meal-related proteins. NCBI reference sequence are marked purple.

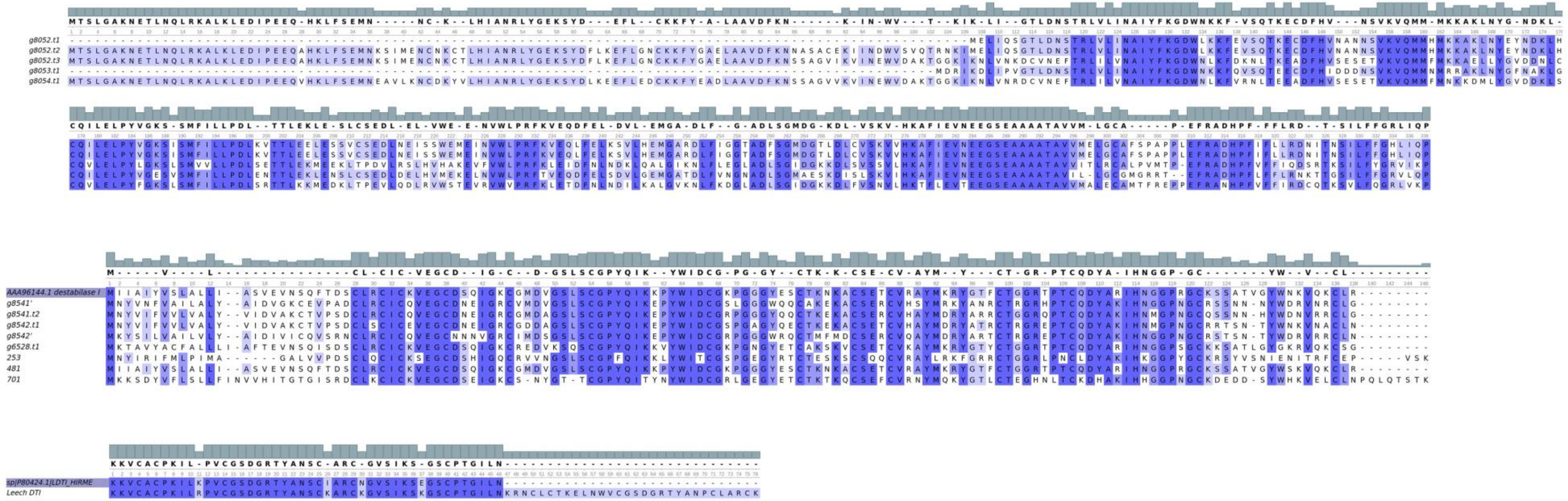

Supplementary Fig. 2. The protein alignment (with percentage Identity coloring scheme) of new homologs of genes encoding known anticoagulants or blood meal-related proteins. NCBI reference sequence are marked purple.

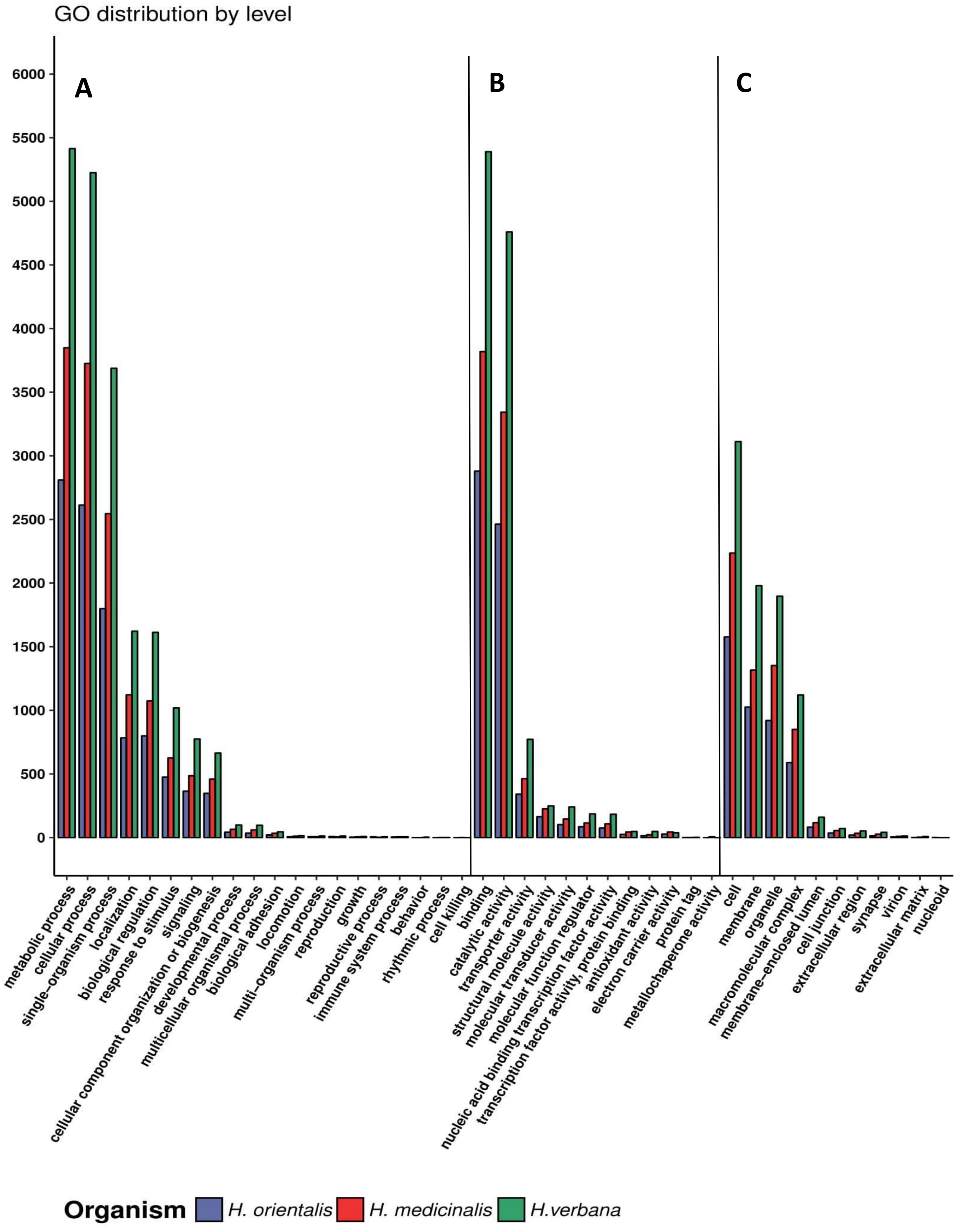

Supplementary Fig. 3. Transcript distribution among functional categories: (a) biological process, (b) molecular function and (c) cellular component.

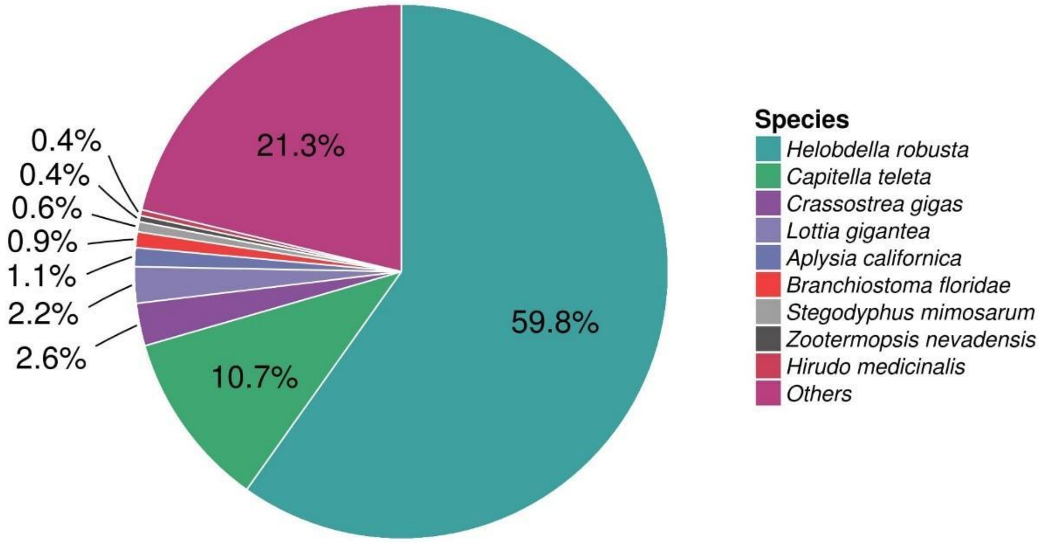

Supplementary Fig. 4. The distribution of the top BLAST hits for all transcriptomes.

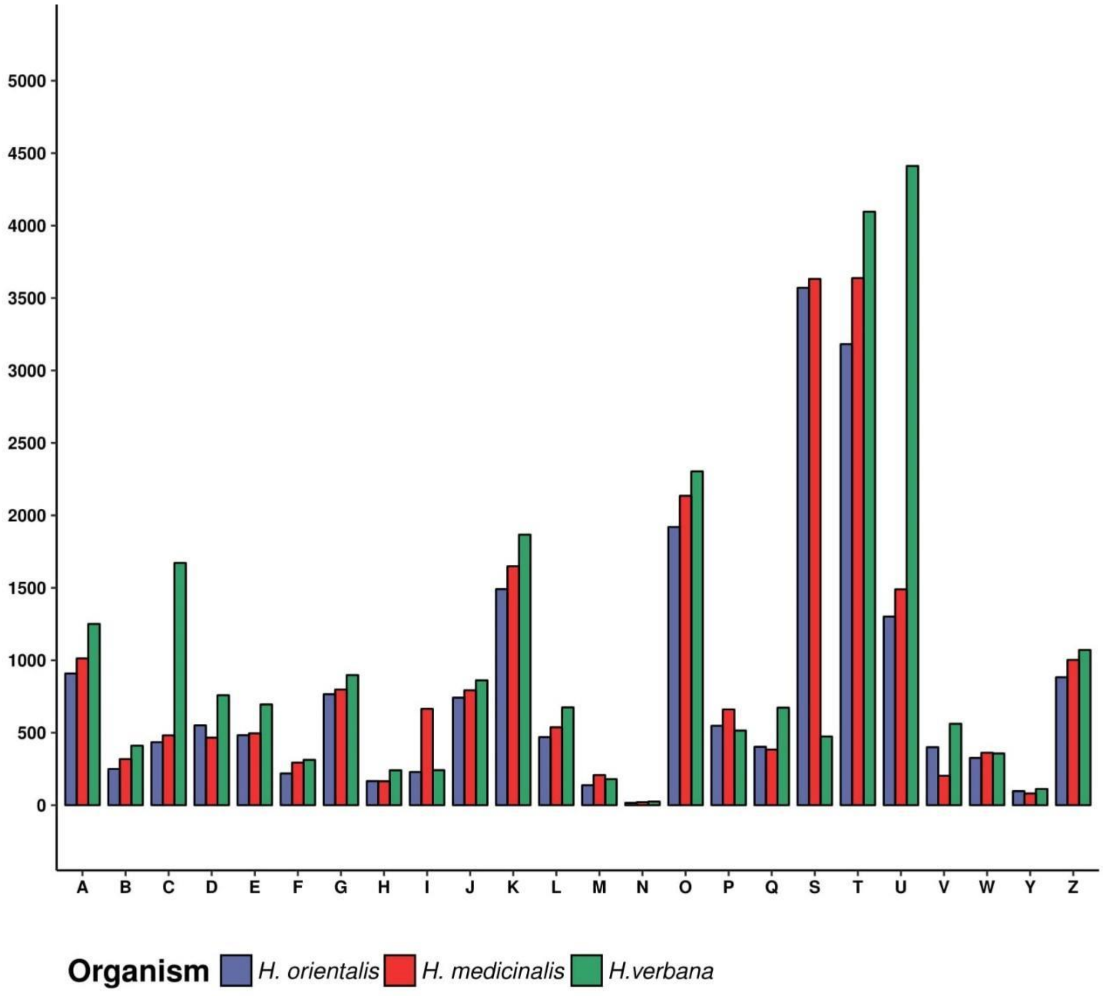

Supplementary Fig. 5. Transcript distributions of three medicinal leech species between KOG categories A, RNA processing and modification; B, chromatin structure and dynamics; C, energy production and conversion D, cell cycle control, cell division, chromosome partitioning; E, amino acid transport and metabolism; F, nucleotide transport and metabolism; G, carbohydrate transport and metabolism; H, coenzyme transport and metabolism; I, lipid transport and metabolism; J, translation, ribosomal structure and biogenesis; K, transcription; L, replication, recombination and repair; M, cell wall/membrane/envelope biogenesis; N, cell

motility; O, posttranslational modification, protein turnover, chaperones; P, inorganic ion transport and metabolism; Q, secondary metabolites biosynthesis, transport and catabolism; R, general function prediction only; T, signal transduction mechanisms; U, intracellular trafficking, secretion, and vesicular transport; V, defense mechanisms; W, extracellular structures; Y, nuclear structure; Z, cytoskeleton; S, function unknown.

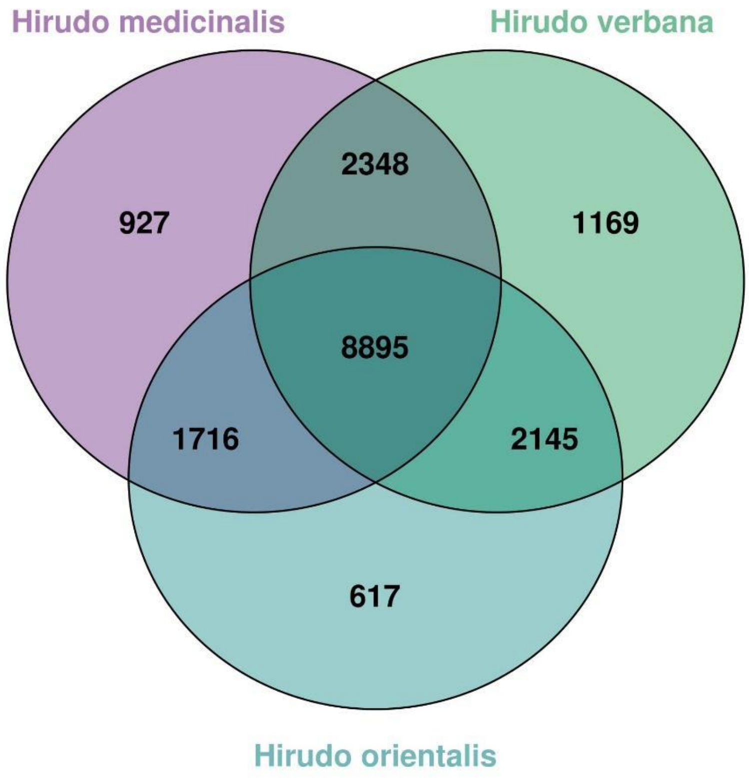

Supplementary Fig. 6. Venn diagram showing numbers of detected orthologous CDS sequences for the transcriptomes of three medicinal leech species.

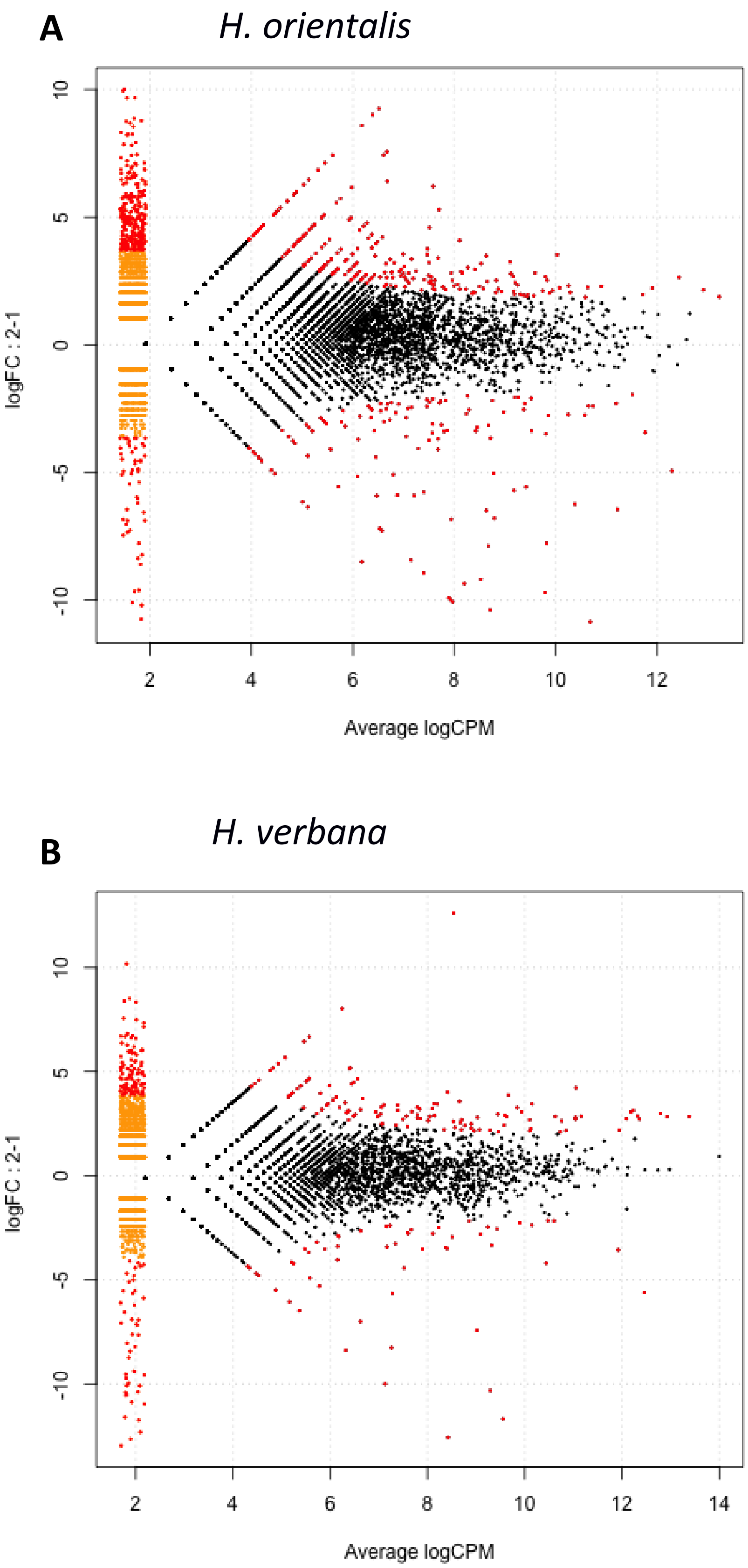

Supplementary Fig.7. MA plots of differentially expressed genes (red dots) in salivary cells and muscles for *H. orientalis* (a) and *H. verbana* (b). MA-plots representing the log Fold Change (logFC) against the log-average log CPM per each transcript cluster across each pair of compared samples (muscle and salivary cells). Differentially expressed clusters supported by FDR < 0.05 are plotted in red.

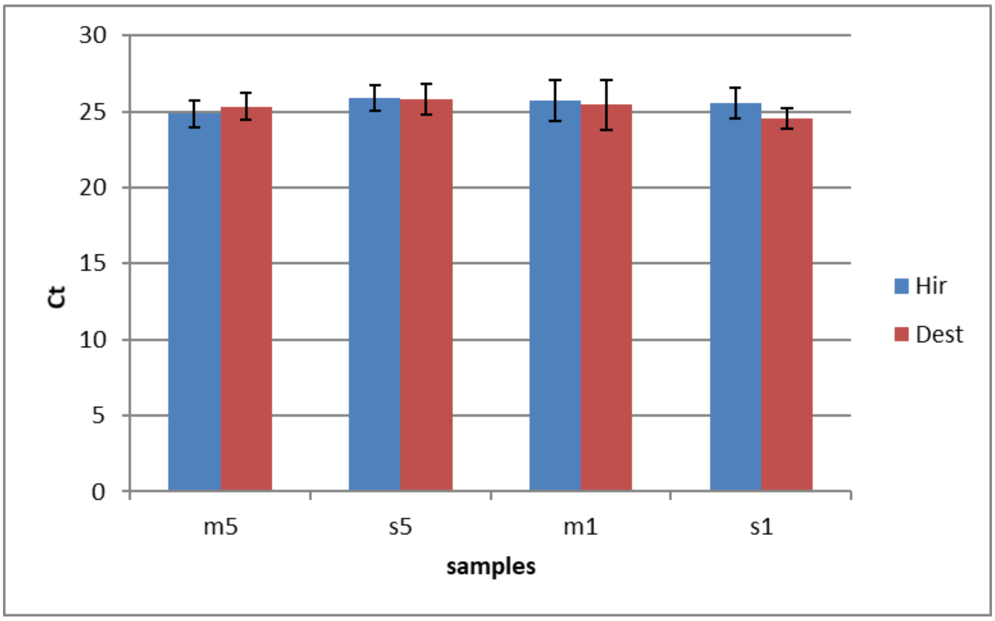

Supplementary Fig. 8. The bar chart shows the mean values of Ct and standard deviations after normalization by the reference gene (tubulin) for target genes (blue - Hirudin and red - Destabilase). RNA for analysis was obtained from samples of LMD sections of muscle tissue and salivary cells. Samples differ in the amount of tissue was cut.

Sample legend:

m5 - 5 LMD sections of muscle tissue

s5 - 5 LMD sections salivary cells

m1- 1 LMD sections of muscle tissue

s1 - 1 LMD sections salivary cells

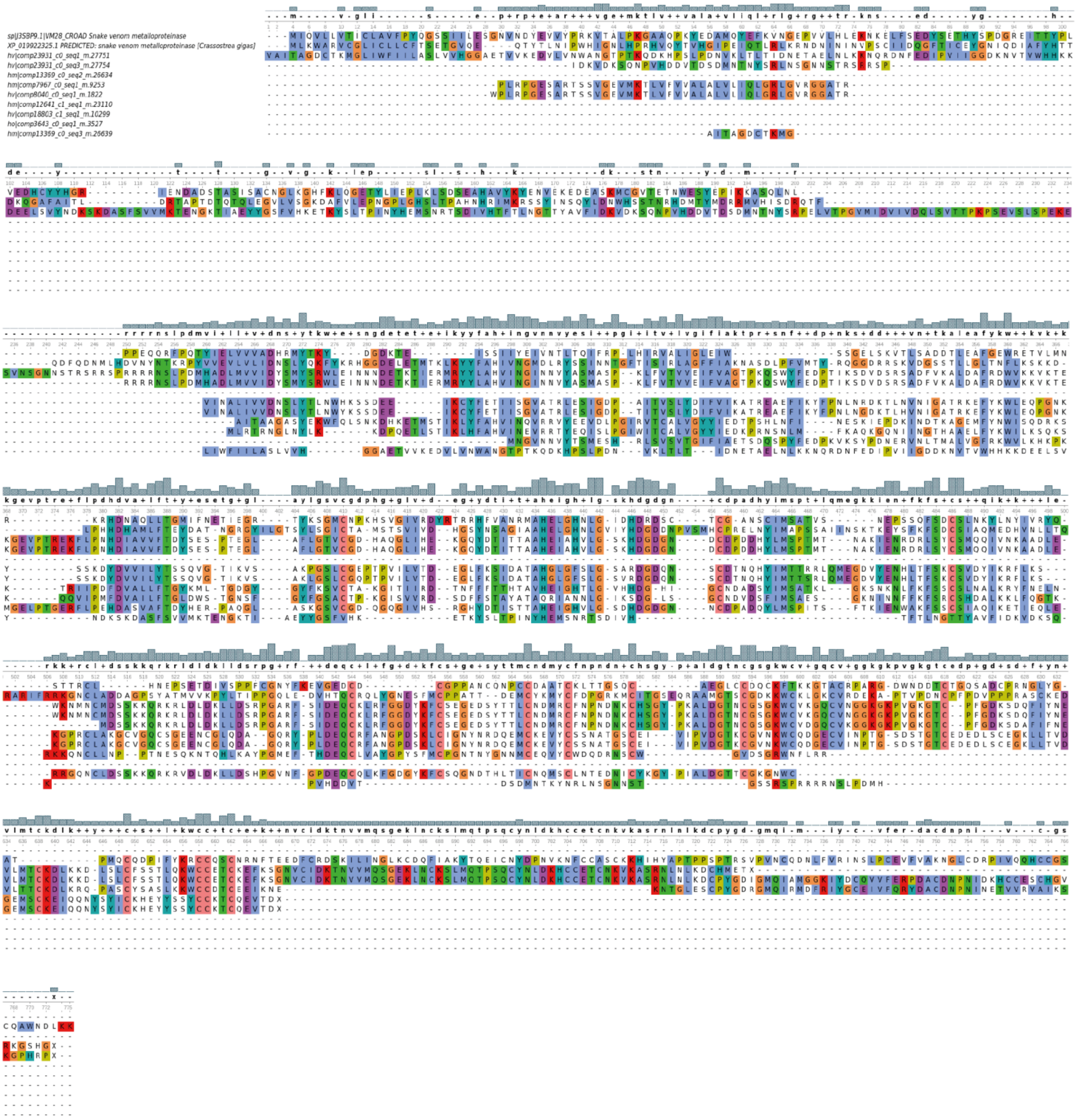

Supplementary Fig. 9. Alignment of M12 domains peptidases with the snake metalloproteinase and mollusk snake metalloproteinase homolog (*Crassostrea gigas*). Alignment generated by muscle with Clustal X Colour Scheme.

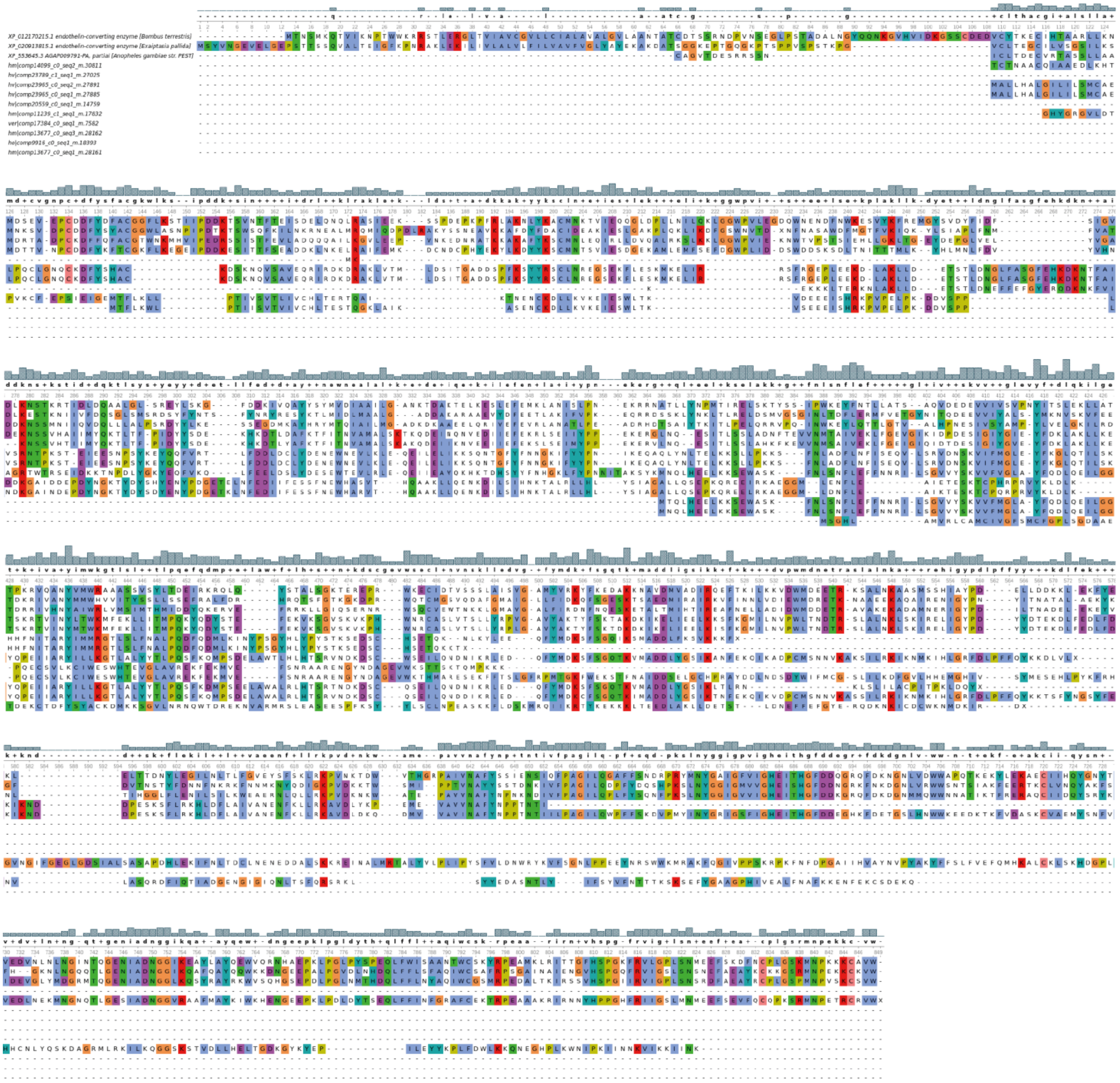

Supplementary Fig. 10. Alignment of M13 domains peptidases with the Bumblebee, sea anemone endothelial-converting enzyme and mosquito homolog. Alignment generated by muscle with Clustal X Colour Scheme.

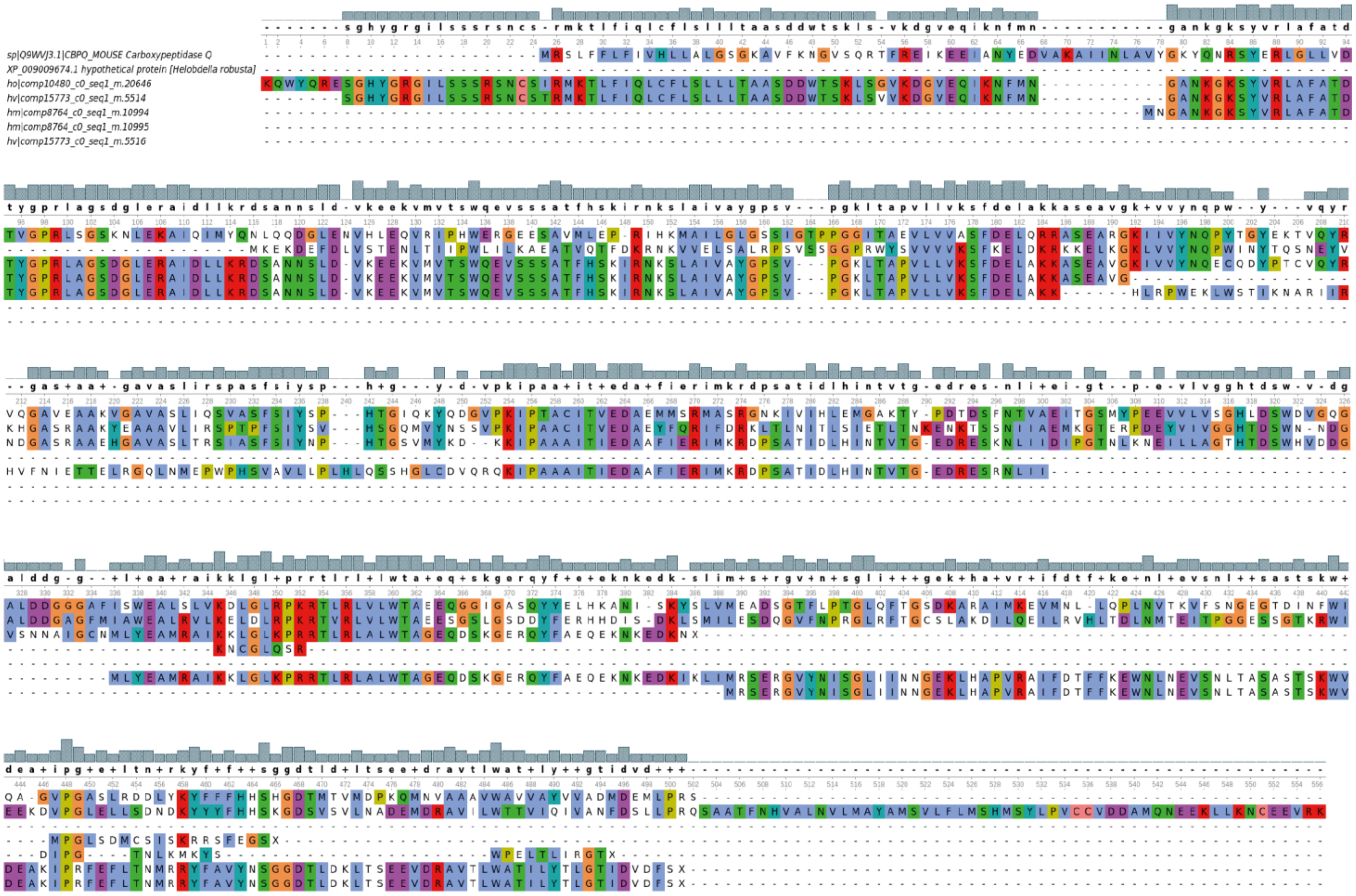

Supplementary Fig. 11. Alignment of M28 domains peptidases with the mouse Q-type carboxypeptidase, and leech (*Helobdella robusta*) homolog. Alignment generated by muscle with Clustal X Colour Scheme.

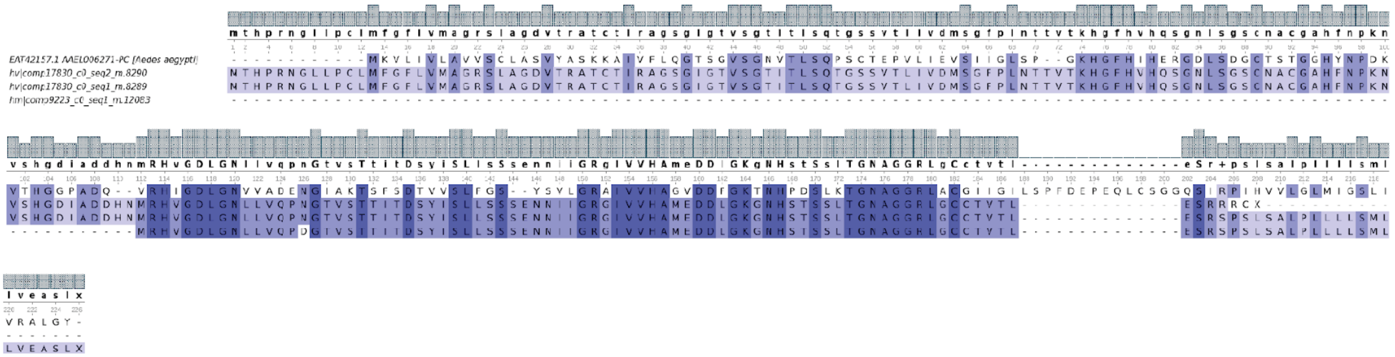

Supplementary Fig. 12. Alignment of Superoxide dismutase domains with the mosquito homolog. Alignment generated by muscle with percentage Identity coloring scheme.

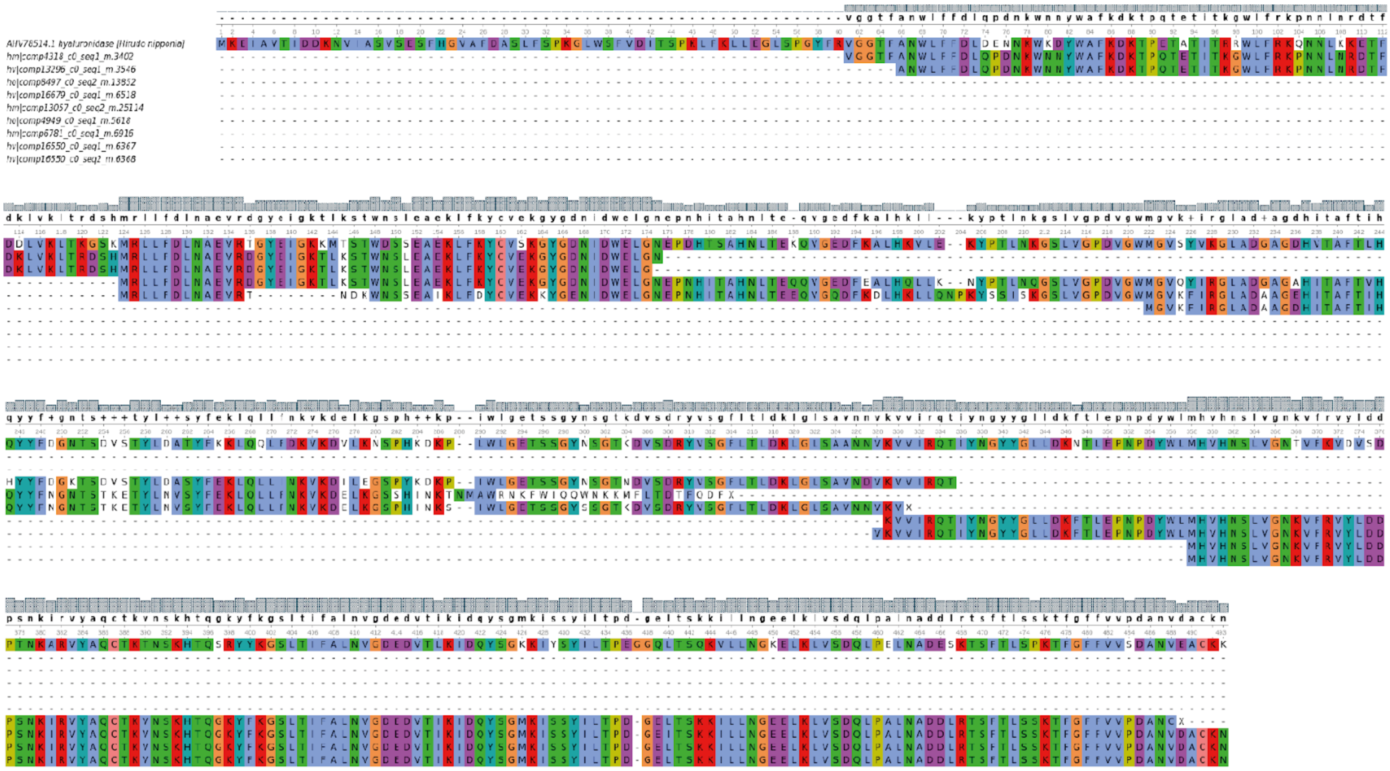

Supplementary Fig. 13. Alignment of Hyaluronidase domains with leech hyaluronidase (*Hirudo nipponia*). Alignment generated by muscle with Clustal X Colour Scheme.

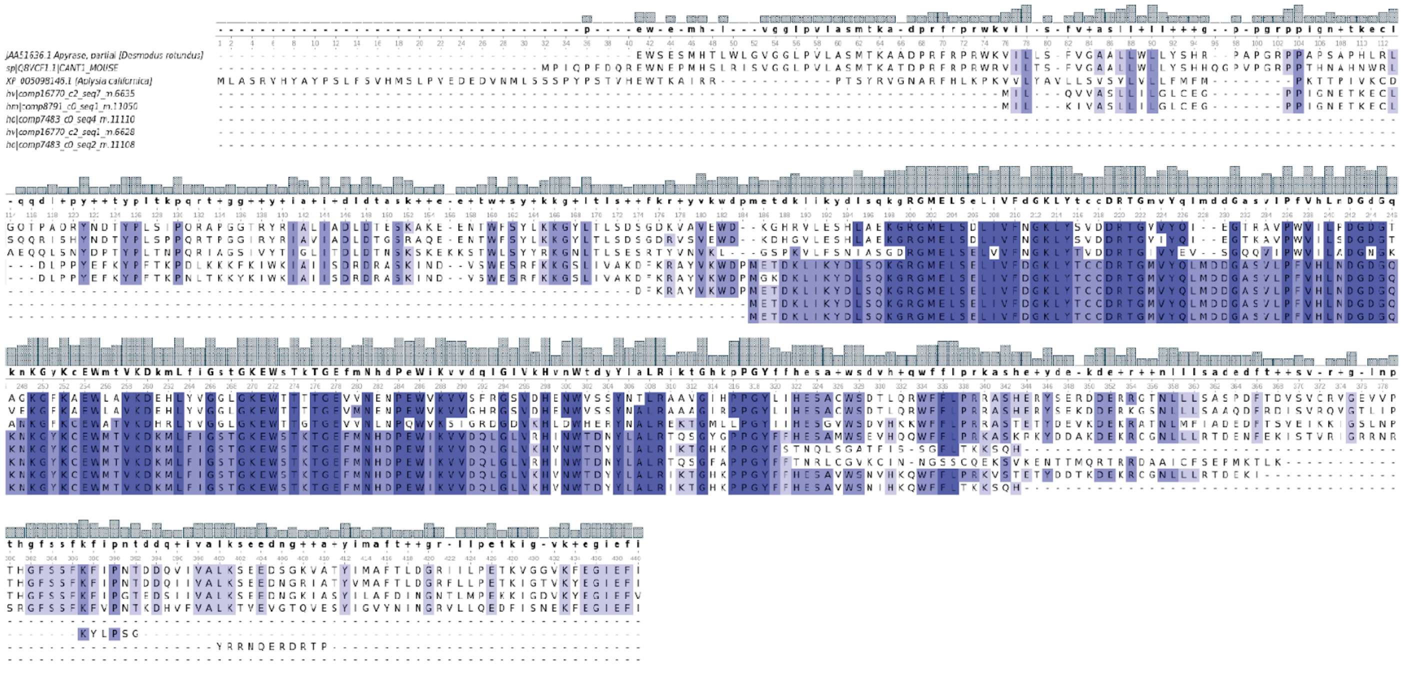

Supplementary Fig. 14. Alignment of Apyrase domains with the vampire bat (*Desmodus rotundus*), mouse Apyrases and mollusk (*Aplysia californica*) homolog. Alignment generated by muscle with percentage Identity coloring scheme.

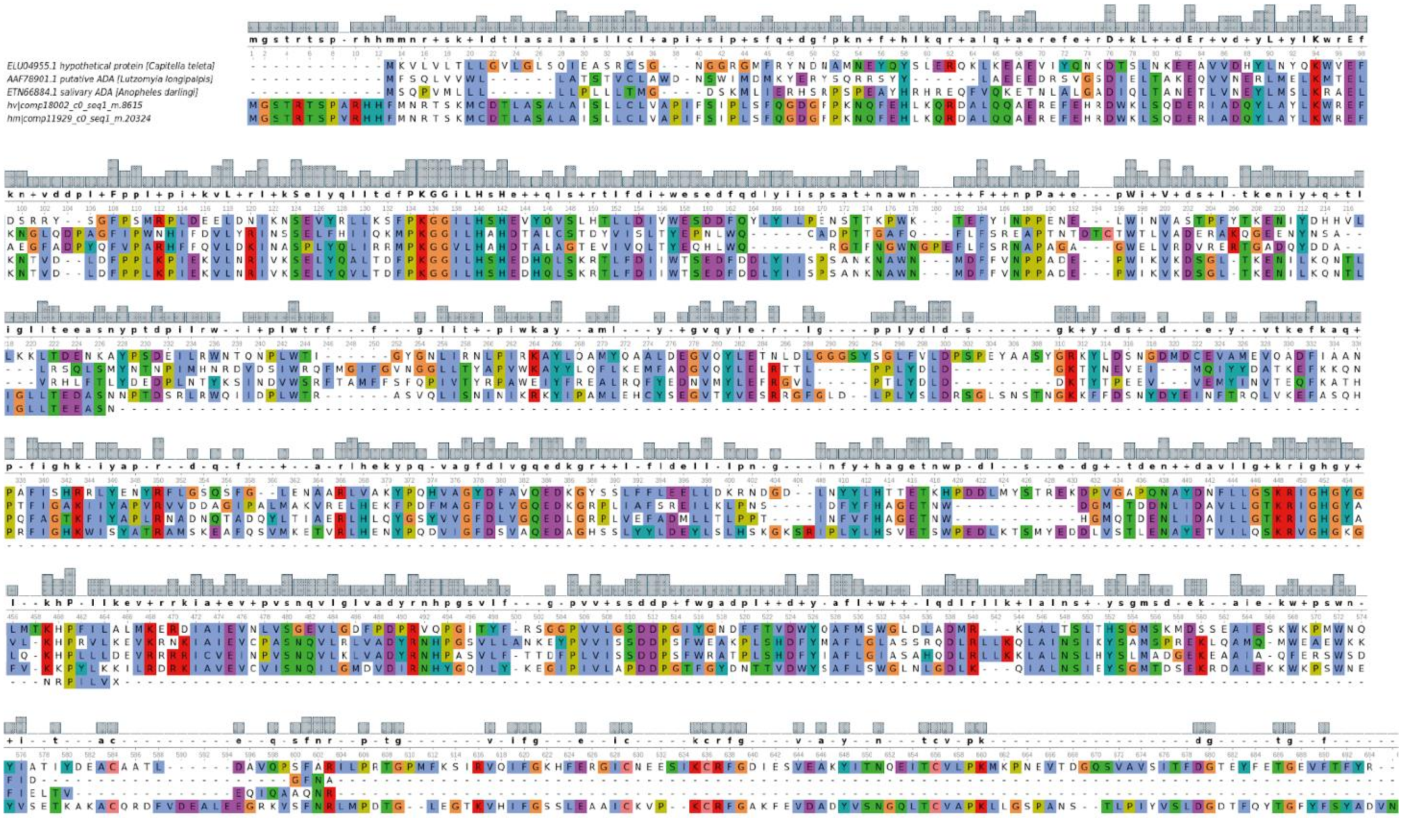

Supplementary Fig. 15. Alignment of Adenosine/AMP deaminase (ADA) domains with mosquitos ADA and homolog from annelid worm (*Capitella teleta*). Alignment generated by muscle with Clustal X Colour Scheme.

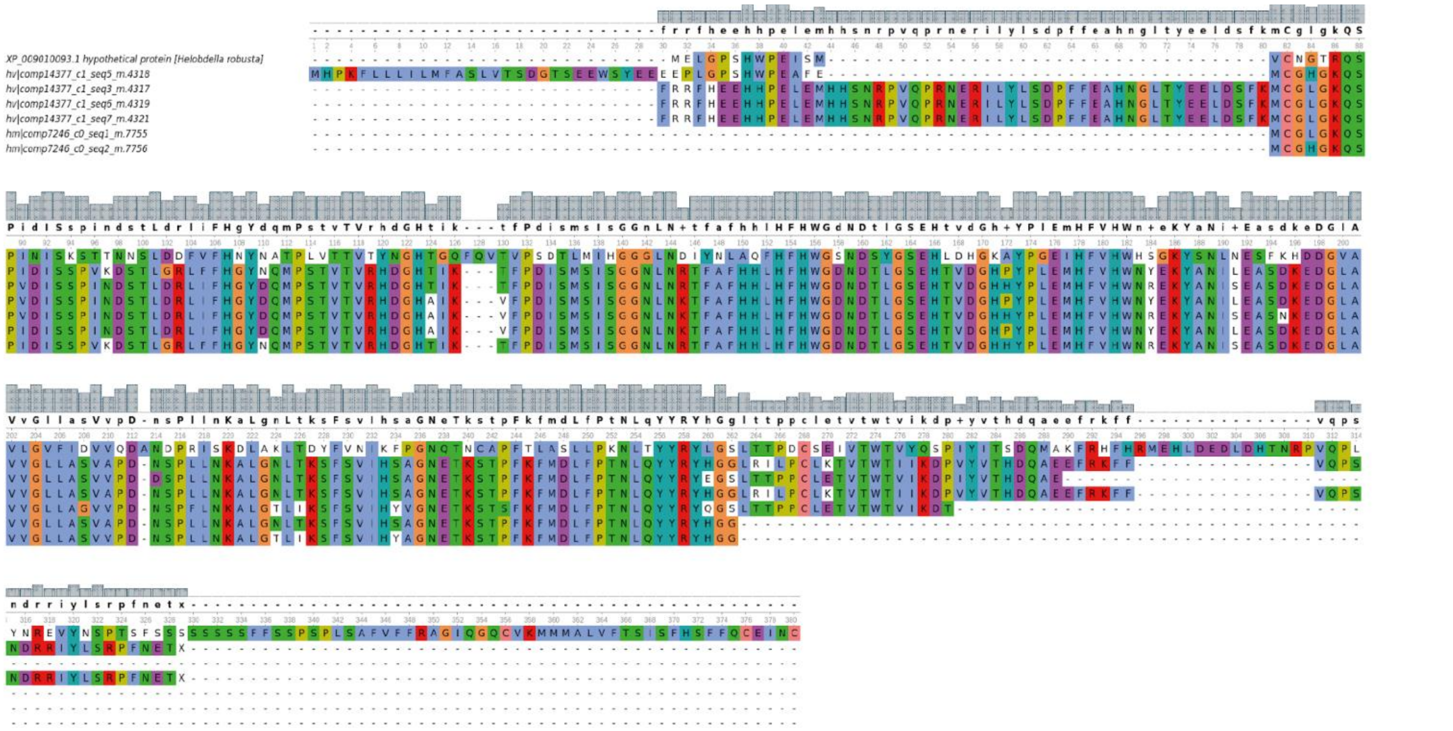

Supplementary Fig. 16. Alignment of Carbonic anhydrase domains with leech (*Helobdella robusta*) homolog. Alignment generated by muscle with Clustal X Colour Scheme.

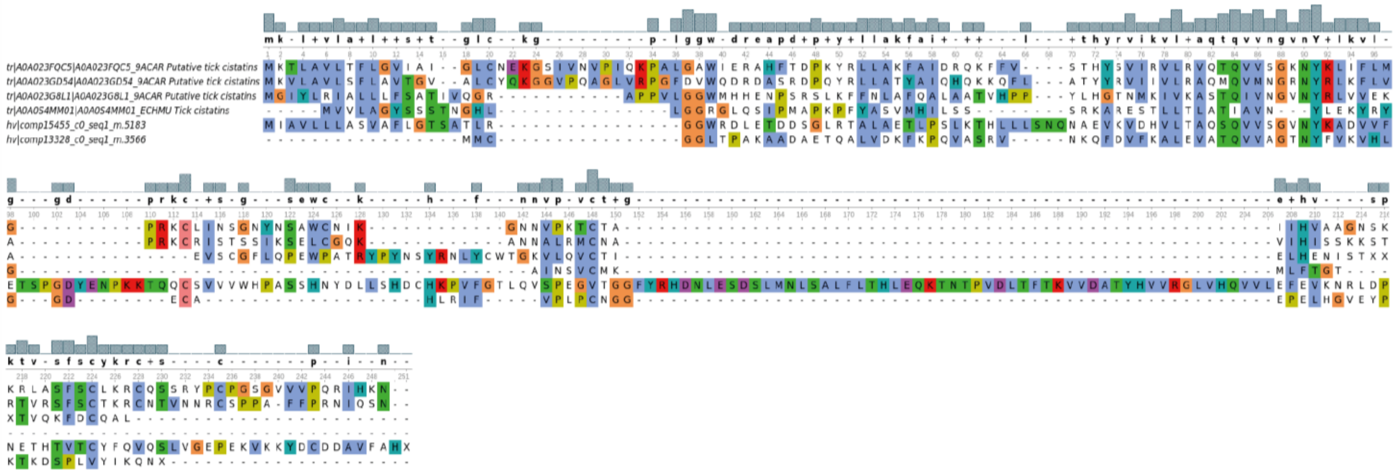

Supplementary Fig. 17. Alignment of Cystatin domains with ticks homologs. Alignment generated by muscle with Clustal X Colour Scheme.

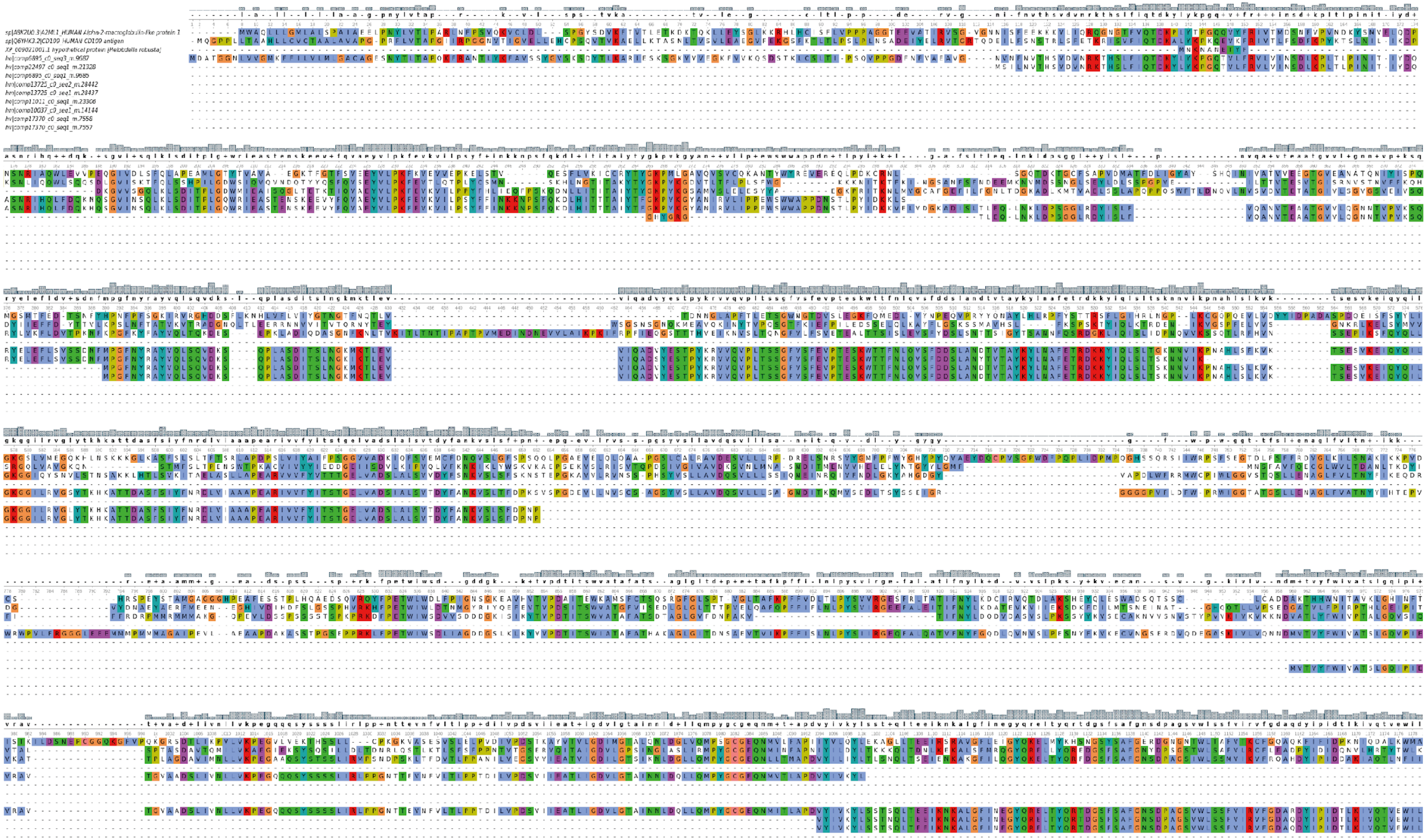
sp|A8K2U0.3|A2ML1_HUMAN Alpha-2-macroglobulin-like protein 1
sp|Q6YHK3.2|CD109_HUMAN CD109 antigen
XP_009021001.1 hypothetical protein [Helobdella robusta]
ho|comp6895_c0_seq3_m.9687
hv|comp22497_c0_seq1_m.21328
ho|comp6895_c0_seq1_m.9685
hm|comp13725_c0_seq2_m.28442
hm|comp13725_c0_seq1_m.28437
ho|comp11011_c0_seq1_m.23306
hm|comp10037_c0_seq1_m.14144
hv|comp17370_c0_seq1_m.7558
hv|comp17370_c0_seq1_m.7557

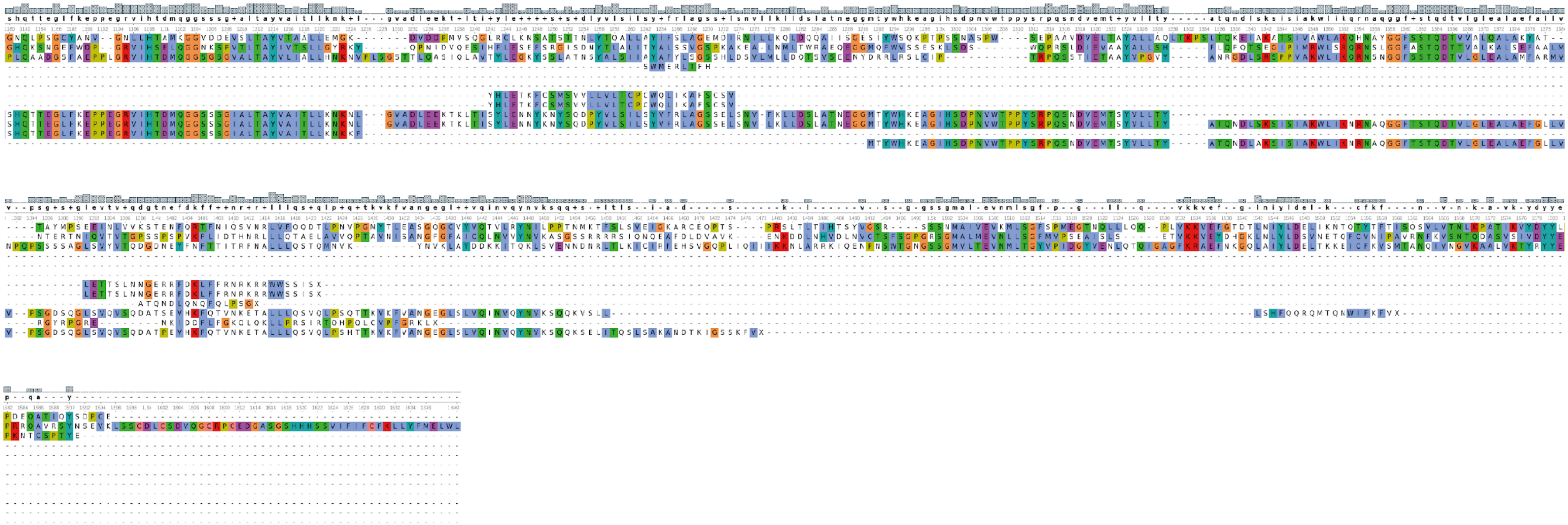

Supplementary Fig. 18. Alignment of Alpha-2-macroglobulin (α2M) domains with human α2M, CD109 and leech (*Helobdella robusta*) homolog. Alignment generated by muscle with Clustal X Colour Scheme.

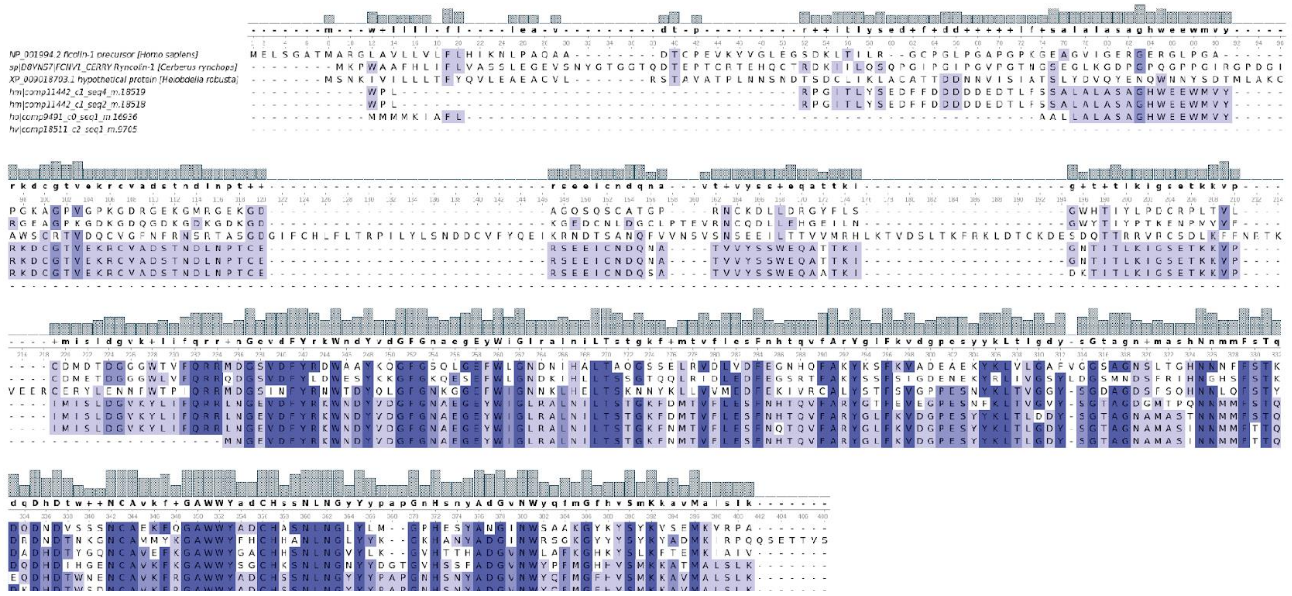

Supplementary Fig. 19. Alignment of Ficolin domains with the human, snake Ryncolin-1 (*Cerberus rynchops*) and leech (Helobdella robusta) homolog. Alignment generated by muscle with percentage Identity coloring scheme.

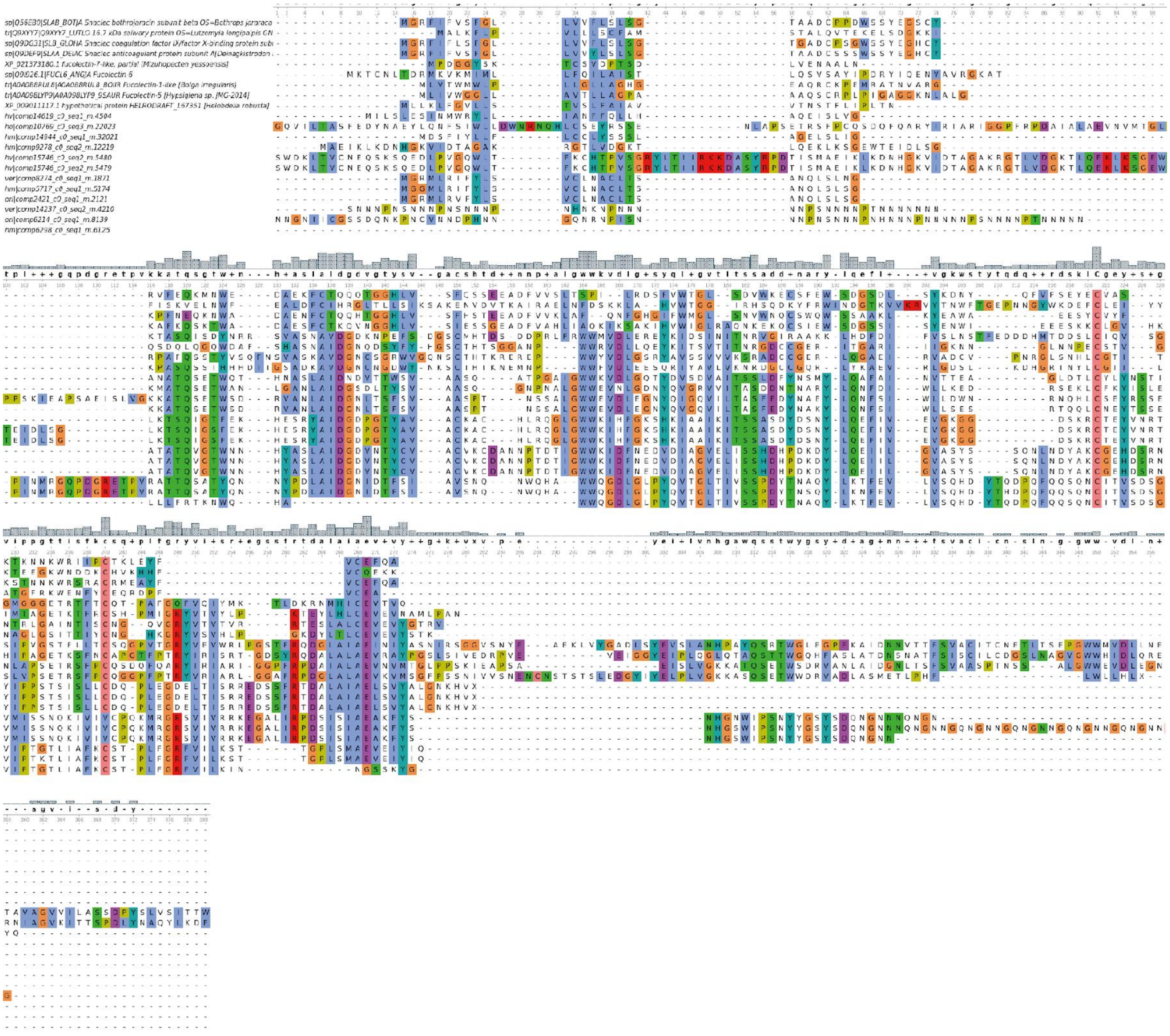

Supplementary Fig. 20. Alignment of F5/8 type C domains with different snaclecs and fucolectins. Alignment generated by muscle with Clustal X Colour Scheme.

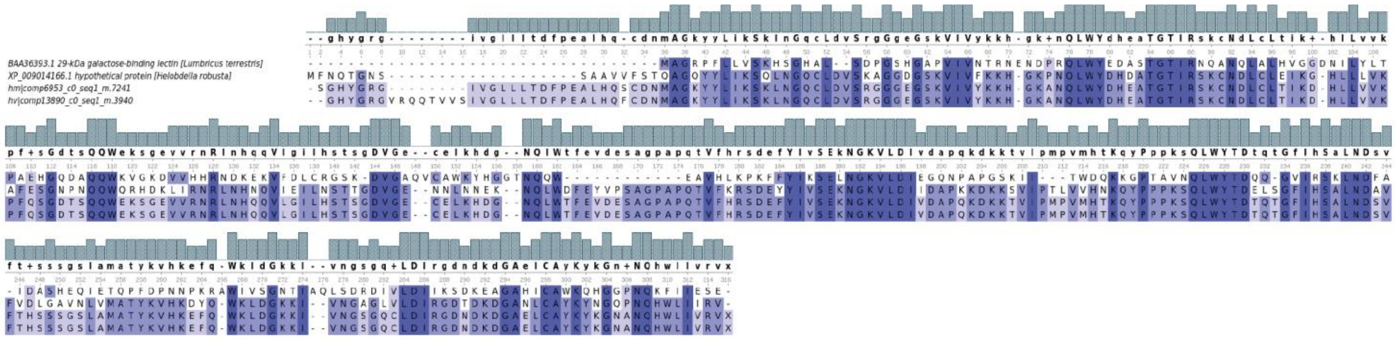

Supplementary Fig. 21. Alignment of Ficolin domains with galactose-binding lectin from *Lumbricus terrestris* and leech (*Helobdella robusta*) homolog. Alignment generated by muscle with percentage Identity coloring scheme.

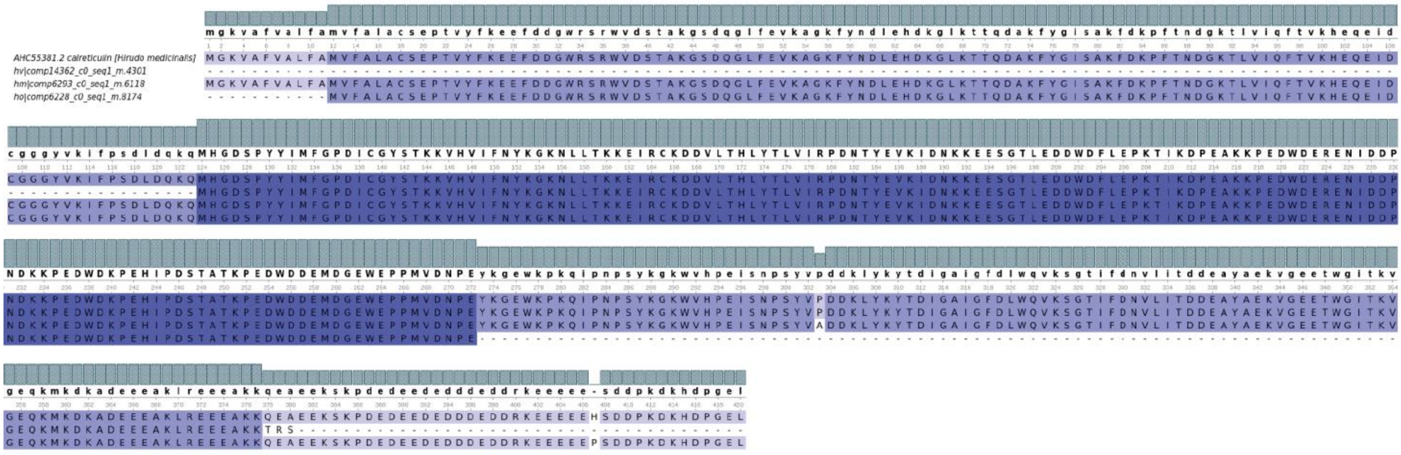

Supplementary Fig. 22. Alignment of Calreticulin domains with calreticulin from *H.medicinalis.* Alignment generated by muscle with percentage Identity coloring scheme.

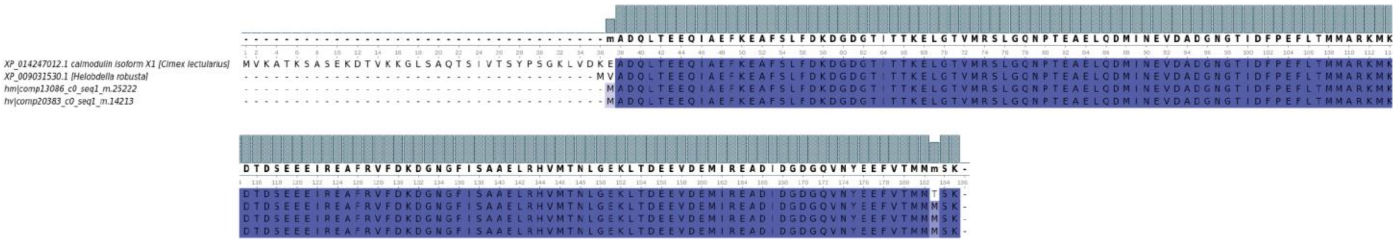

Supplementary Fig. 23. Alignment of Calmodulin domains with the tick calmodulin and leech (*Helobdella robusta*) homolog. Alignment generated by muscle with percentage Identity coloring scheme.

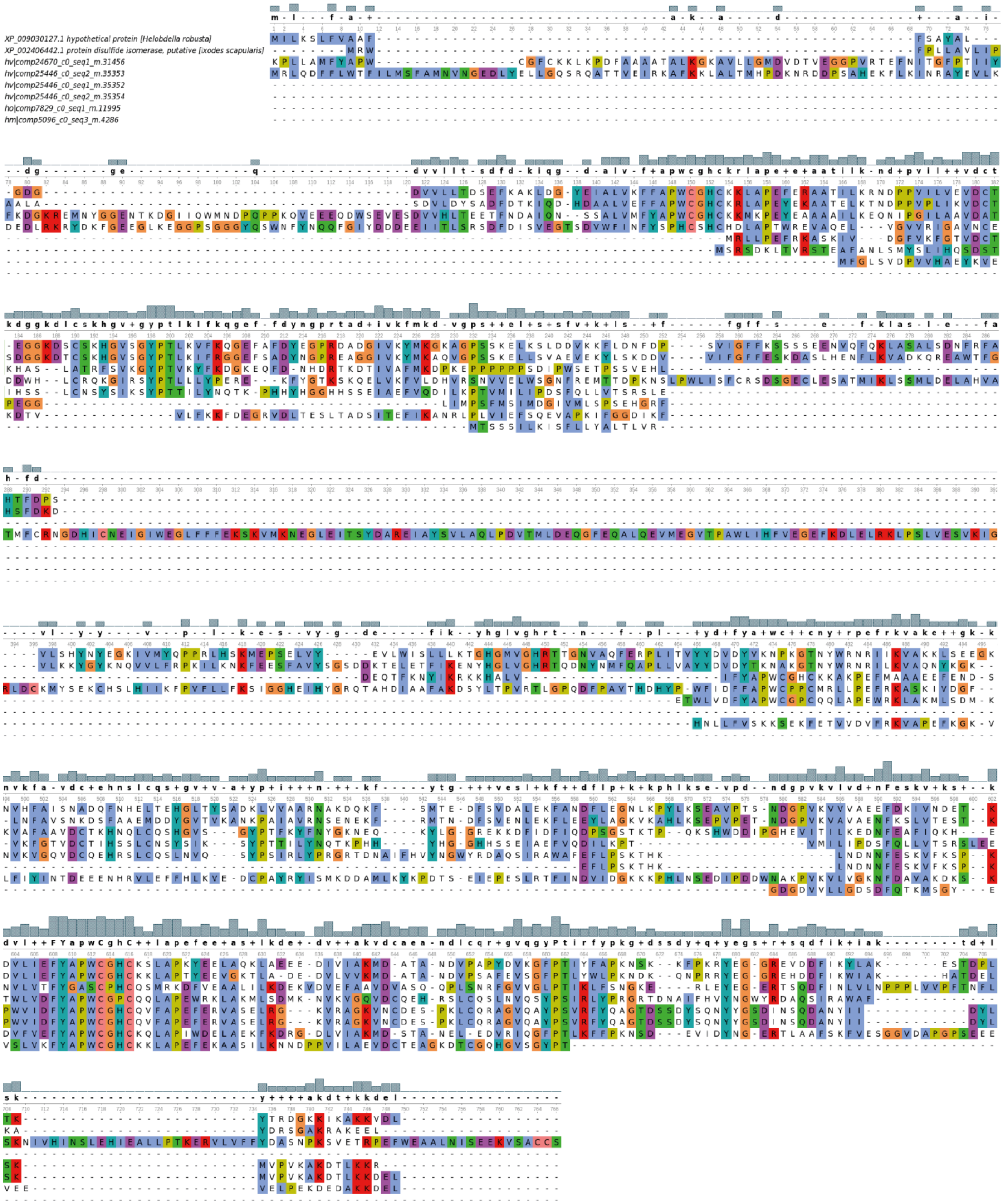

Supplementary Fig. 24. Alignment of Thioredoxin-like domains with tick disulfide isomerase and leech (*Helobdella robusta*) homolog. Alignment generated by muscle with Clustal X Colour Scheme.

Supplementary Tables

Supplementary Table 1. Description of libraries used for genome assembly.

*Read type: SE – single end, PE – pair end, MP –mate pair

**Ion Proton**				**#SRR6926478**
read lenght	***read type****	***total reads***	***Total Mb***	***coverage***
200bp	SE	47,502,374	11,292	x50
**Ion Torrent, True Mate insert size 5kbp**				**#SRR6926477**
400bp	MP	1,416,496	512	x2,5
**Illumina MiSeq, Nextera mate pair insert size 10kbp**				**#SRR6926476**
150bp	SE	150,286	328	x1,5
300bp	PE	6,161,612	1,955	x9
300bp	MP	6,953,316	2,208	x10

Supplementary Table 2. SPAdes genome assembly statistics

**Sum (bp)**	232,003,367
**Total number of contigs**	168,624
**N50**	12,947
**Max contig size**	1,675,099
**Min contig size**	128

Supplementary Table 3. Binning statistics

	**host**	**bacteria**
**Sum (bp)**	154,802,040	48,830,001
**Total number of contigs**	29,764	4,094
**N50**	13,786	31,787
**Max contig size**	188,484	1,675,099
**Min contig size**	500	500

Supplementary Table 4. Eukaryotic contigs scaffolding statistics (Sspase)

**Sum (bp)**	187,580,802
**Total number of scaffolds**	14,042
**N50**	97,775
**Max scaffold size**	920,285
**Min scaffold size**	500
**Average scaffold size**	13,358
**Total number of N's**	32,778,762
**Sum (bp) no N's**	154,802,040

Supplementary Table 5. *Hirudo medicinalis* genome annotation statistics

Characteristic	*Count*
Total sequence length	187580802
Number of genes	14596
Number of mRNAs	15728
Number of exons	129359
Number of introns	113631
Number of CDS	15728
Overlapping genes	389
Contained genes	66
CDS: complete	15728
CDS: start, no stop	0
CDS: stop, no start	0
CDS: no stop, no start	0
Total gene length	102198381
Total mRNA length	110044980
Total exon length	28951546
Total intron length	81320696
Total CDS length	21901875
Shortest gene	255
Shortest mRNA	255
Shortest exon	3
Shortest intron	42
Shortest CDS	201
Longest gene	111002
Longest mRNA	111002
Longest exon	10212
Longest intron	42547
Longest CDS	26013
mean gene length	7002
mean mRNA length	6997
mean exon length	224
mean intron length	716
mean CDS length	1393
% of genome covered by genes	54,5
% of genome covered by CDS	11,7
mean mRNAs per gene	1
mean exons per mRNA	8
mean introns per mRNA	7

Supplementary Table 6. Size and distribution of DNA on *Hirudo medicinalis* genome scaffolds.

Scaffold Length	No.Scaffolds	Total Bases	% Genome in Scaffolds*
500kb-1Mb	10	6,372,431	2,896559545
100kb-499kb	459	85,603,941	38,91088227
10kb-99kb	2,085	72,201,282	32,81876455
<10kb	11,488	23,403,148	10,63779455
**total**	**14,042**	**187,580,802**	**85,26400091**

*Calculations are based on the genome size estimate of 220 Mb
http://www.genomesize.com/result_species.php?id=1061

Supplementary Table 7. Blood meal related genes and genes encoding known anticoagulants. Reference genes are colored blue. Genes identified without the use of automatic annotation colored red. Known anticoagulants and products of blood meal related genes identified in transcriptomic data.

Type Molecule	Pfam/ InterPro	gene id	scaffold id	start	stop	gene size (bp)	№ exons	Transcript Length (bp)	Lenght (aa)	transcript id
Bdellin A/ Hirustasin	PF02822 (*Antistasin*)	**g11304.t1**	scaffold1202|size 46802	20181	23845	3664	4	711	237	-
		**g11304'**	scaffold1202|size 46802	22441	23034	593	4	-	-	comp8393_c0_seq1| m.10226
		**g11305.t1**	scaffold1202|size 46802	25435	29835	4400	4	492	164	-
		**g11305'**	scaffold1202|size 46802	-	-	-	4	-	-	-
		**g11305***	scaffold1202|size 46802	-	-	-	4	-	-	-
		**g11306.t1**	scaffold1202|size 46803	41426	46622	5196	6	1167	389	-
		g4529.t1	scaffold210|size3 45909	124016	125423	1407	7	399	133	-
		g6012.t1	scaffold335|size6 3276	54026	62233	8207	5	318	106	-
Antistasin like	PF02822 (*Antistasin*)	**g4239.t1**	scaffold188|size6 7202	9556	12796	3240	10	1173	391	comp11393_c0_seq1| m.18362
Bdellin B3	PF00050 (*Kazal-type serine protease inhibitor domain*)	**g6343.t1**	scaffold364|size2 17826	196186	197474	1288	3	561	187	comp5703_c0_seq2| m.5161
		**g6344.t1**	scaffold364|size2 17827	197994	199424	1430	3	747	249	-
		**g6345.t1**	scaffold364|size2 17827	199887	201284	1397	3	912	304	comp5677_c0_seq1| m.5126
		**g6346.t1**	scaffold364|size2 17828	202703	204214	1511	3	861	287	-
		**g6347.t1**	scaffold364|size2 17829	206025	207066	1041	3	513	171	-
		**g6348.t1**	scaffold364|size2 17828	208945	210916	1971	3	774	258	comp7978_c0_seq1| m.9276
Eglin-C	PF00280 (*Potato inhibitor I family)*	**g11560.t1**	scaffold1303|size 13825	1414	5265	3851	3	774	258	comp5230_c0_seq2| m.4481
		**g11560'**	scaffold1303|size 13825							-
		**g11561.t1**	scaffold1303|size 13825	5893	9585	3692	4	489	163	-
		**g11561'**	scaffold1303|size 13825	-	-	-	-	-	-	-
		g12915.t1	scaffold2531|size 16879	6063	16605	10542	6	891	297	-
		g11258.t1	scaffold1188|size 60920	4236	5605	1369	5	603	201	-
		g11720.t1	scaffold1379|size 30790	2356	5157	2801	5	915	305	-
Lectoxin	PF00059 (*Lectin C-*	**g11865.t1**	scaffold1454|size 22262	8296	10271	1975	5	1386	462	comp7752_c0_seq2| m.8823

	type *domain*)	g13772.t1	scaffold4397|size 2052	576	1775	1199	3	759	253	-
		g12022.t1	scaffold1543|size 37248	31836	32983	1147	3	807	269	comp6281_c0_seq1| m.6094
**Saratin/ LAPP**	NA	**g9274.t1**	scaffold725|size8 2469	58496	60431	1935	4	633	211	comp9252_c0_seq4| m.12147
		g6796.t1	scaffold404|size7 0608	56461	58105	1644	4	705	235	-
**Carboxy peptidase inhibitor**	IPR024063 (*Proteinase inhibitor I46*)	**g7223.t1**	scaffold451|size2 23322	71666	72309	643	3	504	168	comp6493_c0_seq2| m.6398
**Hirudins**	PF00713 (*Hirudin*)	**g9136.t1**	scaffold705|size2 26376	6617	7515	898	4	384	128	-
		hirudinII	scaffold2168|size 5612	121	5347	5226	-	-	-	-
**Hirudin-like factor**	IPR011061 (*Hirudin/ antistatin*)	**g9138.t1**	scaffold705|size2 26377	12662	13565	903	4	318	106	-
		**g9139.t1**	scaffold705|size2 26378	14431	16375	1944	5	861	287	-
**Elastase inhibitor**	PF00079 (*Serpin*)	g8052.t1	scaffold551|size1 29727	90668	95785	5117	1	1206	402	comp13340_c0_seq2| m.26472
		g8052.t2	scaffold551|size1 29728	90668	95785	5117	3	1593	531	comp13340_c0_seq5| m.26478
		g8052.t3	scaffold551|size1 29729	93413	95785	2372	3	1869	623	comp12937_c0_seq1| m.24474
		g8053.t1	scaffold551|size1 29730	102789	103805	1016	1	1017	339	comp13340_c0_seq4| m.26475
		g8054.t1	scaffold551|size1 29731	104600	109715	5115	4	1995	665	comp12937_c0_seq2| m.24476
**Destabilase**	PF05497 (*Destabilase*)	g6528.t1	scaffold380|size1 14101	66756	67848	1092	4	627	209	comp6811_c0_seq1| m.6970
		**g8541’**	scaffold619|size5 4468	39834	38945	890	4	408	136	-
		**g8541.t2**	scaffold619|size5 4468	29596	35082	5486	4	570	190	comp8255_c0_seq1| m.9920
		**g8542.t1**	scaffold619|size5 4469	44785	53915	9130	4	672	224	-
		**g8542’**	scaffold619|size5 4468	53885	52779	1107	4	408	136	-
		253	scaffold253|size1 72121	22531	20524	2008	4	405	135	-
		481	scaffold481|size5 2494	8363	9924	1562	4	339	113	-
		701	scaffold701|size2 61053	52345	46410	5936	3	384	128	-
**Cystatin**	PF00031 (*Cystatin domain*)	g11713.t1	scaffold1375|size 41249	24999	25915	916	3	675	225	comp4284_c0_seq1| m.3360
		g10530.t1	scaffold991|size3 4421	4186	6605	2419	4	627	209	comp8165_c0_seq1| m.9682
		g9499.t3	scaffold770|size1 10308	27016	30656	3640	6	1110	370	comp11027_c0_seq1| m.17173; comp11027_c0_seq7_ m.17184
**Leech-derived tryptase inhibitor C**	PF00050 (*Kazal-type serine protease inhibitor domain*)	Leech DTI	scaffold11|size16 5743	8937	9555	618	-	-	-	-

Supplementary Table 8. IonTorrent libraries used in transcriptome assembly.

species	sample type	nucleotides	≥ Q20	Total reads	mean read length	SRA
Hirudo medicinalis	salivary gland	252,504,386	210,313,049	980,679	257	SRR6929412
	muscles	250,690,103	209,337,376	977,623	256	SRR6929413
	salivary gland norm	430,515,097	345,443,320	2,180,832	197	SRR6929414
	muscles norm	538,447,451	428,865,131	2,736,026	196	SRR6929411
Hirudo verbana	salivary gland	258,669,154	204,094,499	1,087,218	237	SRR6929409
	muscles	266,320,536	206,481,733	1,113,457	239	SRR6929410
	salivary gland norm	535,151,381	444,198,915	2,580,419	207	SRR6929407
	muscles norm	615,733,423	507,545,320	2,779,310	221	SRR6929408
Hirudo orientalis	salivary gland	287,546,083	218,597,170	1,154,562	249	SRR6929415
	muscles	281,748,372	215,732,664	1,149,725	245	SRR6929416
	salivary gland norm	423,985,968	345,817,680	2,231,299	200	SRR6929417
	muscles norm	327,703,608	255,928,151	2,230,625	150	SRR6929418

Supplementary Table 9. Transcriptome assembly statistics (Trinity)

species	*H. medicinalis*	*H. orientalis*	*H.verbana*
**Counts of transcripts**			
Total trinity 'genes'	22,615	20,506	33,156
Total trinity transcripts	37,546	31,679	60,320
Percent GC	41.73	40.95	40.67
**Stats based on ALL transcript contigs**			
Contig N50	1,188	1,332	997
Median contig length	692	777	453
Average contig	880	977	692
Total assembled bases	33,061,001	30,957,201	41,793,585
**Stats based on ONLY LONGEST ISOFORM per 'GENE'**			
Contig N50	1,255	1,384	1,000
Median contig length	684	744	397
Average contig	894	971	665
Total assembled bases	20,223,126	19,918,312	22,062,102

Supplementary Table 10. Clusters of orthologs identified by transcriptomic analysis in the salivary cells of three medicinal leech species.

claster N	number of orthologs	H.verbana id	H.medicinalis id	H.orientalis id	PFAM/ UniProt id	description	gene model id*	category
**cluster32**	3	hv|comp13454_c0_seq1_m.3646	hm|comp7187_c0_seq2_m.7679	ho|comp6605_c0_seq1_m.9035	PF02822	Antistasin family	g6902.t1	*Antistasin*
**cluster24**	3	hv|comp16770_c2_seq7_m.6635	hm|comp8791_c0_seq1_m.11050	ho|comp7483_c0_seq4_m.11110	PF06079	Apyrase	g4389.t1	*Apyrase*
**cluster103**	2	hv|comp16770_c2_seq1_m.6628	-	ho|comp7483_c0_seq2_m.11108	PF06079	Apyrase	-	*Apyrase*
**cluster38**	3	hv|comp14362_c0_seq1_m.4301	hm|comp6293_c0_seq1_m.6118	ho|comp6228_c0_seq1_m.8174	PF00262	Calreticulin family	g1489.t1	*Calreticulin*
**cluster4**	5	hv|comp15457_c0_seq1_m.5186	hm|comp8795_c0_seq2_m.11067 ; hm|comp8795_c0_seq1_m.11065 ; hm|comp8795_c0_seq3_m.11068	ho|comp7800_c0_seq4_m.11907	N/A	Cysteine-rich secretory protein family	g14584.t1	*CRISP*
**cluster39**	3	hv|comp21924_c0_seq1_m.19085	hm|comp12280_c0_seq1_m.21617	ho|comp10960_c0_seq2_m.22997	PF00008	EGF-like domain	g5065.t1	*EGF-like*
**cluster33**	3	hv|comp8274_c0_seq1_m.1871	hm|comp5717_c0_seq1_m.5174	ho|comp2421_c0_seq1_m.2121	N/A	F5/8 type C domain	g7683.t1	*F5/8 type C domain like*
**cluster44**	3	hv|comp14237_c0_seq2_m.4210	hm|comp6298_c0_seq1_m.6125	ho|comp6214_c0_seq1_m.8139	PF00754	F5/8 type C domain	g7683.t1	*F5/8 type C domain like*
**cluster36**	3	hv|comp18803_c1_seq1_m.10299	hm|comp12641_c1_seq1_m.23110	ho|comp3643_c0_seq1_m.3527	PF01421	Reprolysin (M12B) family zinc metalloprotease	g11594.t1	*protease M12*
**cluster80**	2	hv|comp17384_c0_seq1_m.7582	hm|comp11239_c1_seq1_m.17832	-	PF01401	Angiotensin-converting enzyme	g9033.t1	*protease M13*
**cluster88**	2	hv|comp24670_c0_seq1_m.31456	hm|comp5096_c0_seq3_m.4286	-	PF00085	Thioredoxin	g5715.t1	*thioredoxin-like*
**cluster9**	4	hv|comp25446_c0_seq2_m.35353 ; hv|comp25446_c0_seq1_m.35352 ; hv|comp25446_c0_seq2_m.35354	-	ho|comp7829_c0_seq1_m.11995	PF00085	Thioredoxin	g11509.t1	*thioredoxin-like*
**cluster93**	2	hv|comp17538_c0_seq1_m.7813	-	ho|comp10986_c0_seq1_m.23149	N/A	N/A32	-	*na*
**cluster106**	2	-	hm|comp10844_c0_seq1_m.16589	ho|comp8184_c0_seq1_m.13000	N/A	N/A33	g10903.t1	*na*
**cluster109**	2	-	hm|comp12107_c1_seq1_m.20950	ho|comp10411_c0_seq1_m.20388	N/A	N/A34	g113.t1	*na*
**cluster110**	2	-	hm|comp16888_c0_seq1_m.33161	ho|comp3681_c0_seq1_m.3572	N/A	N/A35	g4541.t2	*na*
**cluster112**	2	-	hm|comp12036_c0_seq6_m.20693	ho|comp7663_c0_seq2_m.11535	N/A	N/A36	g4110.t1	*na*
**cluster115**	2	-	hm|comp11238_c0_seq1_m.17831	ho|comp5634_c0_seq1_m.6892	N/A	N/A37	g4652.t1	*na*
**cluster116**	2	-	hm|comp13250_c0_seq1_m.26023	ho|comp10856_c0_seq2_m.22462	N/A	N/A38	g7682.t1	*na*
**cluster117**	2	-	hm|comp7084_c0_seq2_m.7499	ho|comp7155_c0_seq1_m.10343	N/A	N/A39	g2583.t1	*na*

**cluster119**	2	-	hm|comp5440_c0_seq1_m.4781	ho|comp3640_c0_seq1_m.3521	N/A	N/A40	g3405.t1	*na*
**cluster122**	2	-	hm|comp11588_c0_seq2_m.19081	ho|comp7009_c0_seq3_m.9937	N/A	N/A41	g9628.t1	*na*
**cluster79**	2	hv|comp25239_c0_seq1_m.34289	-	ho|comp3386_c0_seq1_m.3184	N/A	N/A42	g1594.t1	*na*
**cluster83**	2	hv|comp12479_c0_seq1_m.3126	-	ho|comp5771_c0_seq1_m.7223	N/A	N/A43	-	*na*
**cluster91**	2	hv|comp18033_c0_seq2_m.8738	-	ho|comp9556_c0_seq1_m.17143	N/A	N/A44	g8790.t2	*na*
**cluster34**	3	hv|comp12392_c0_seq1_m.3076	hm|comp9145_c1_seq1_m.11901	ho|comp5121_c0_seq1_m.5952	N/A	tN/A2	g5080.t1	*na*
**cluster43**	3	hv|comp17209_c0_seq1_m.7259	hm|comp11339_c1_seq1_m.18153	ho|comp9212_c0_seq1_m.16064	N/A	tN/A3	-	*na*
**cluster108**	2	-	hm|comp3160_c0_seq1_m.2292	ho|comp1084_c0_seq1_m.899	PF00001	7 transmembrane receptor (rhodopsin family)	g11982.t1	*other*
**cluster114**	2	-	hm|comp10201_c0_seq1_m.14640	ho|comp8992_c0_seq1_m.15415	PF00001	7 transmembrane receptor (rhodopsin family)	g11423.t1	*other*
**cluster120**	2	-	hm|comp5355_c0_seq1_m.4683	ho|comp4032_c0_seq1_m.4124	PF01064	Activin types I and II receptor domain	g9152.t1	*other*
**cluster89**	2	hv|comp16321_c1_seq1_m.6134	hm|comp12110_c0_seq7_m.20957	-	PF02267	ADP-ribosyl cyclase/cyclic ADP-ribose hydrolase	g1570.t1	*other*
**cluster111**	2	-	hm|comp10998_c0_seq2_m.17075	ho|comp10206_c0_seq2_m.19572	PF13520	Amino acid permease	-	*other*
**cluster95**	2	hv|comp25133_c1_seq1_m.33724	hm|comp3917_c0_seq1_m.3045	-	PF12796	Ankyrin repeats (3 copies)	g6671.t1	*other*
**cluster99**	2	hv|comp19190_c0_seq1_m.11233	-	ho|comp10237_c0_seq1_m.19705	PF07742	BTG family	-	*other*
**cluster90**	2	hv|comp31452_c0_seq1_m.38818	hm|comp8714_c0_seq1_m.10892	-	PF00028	Cadherin domain	g1296.t1	*other*
**cluster118**	2	-	hm|comp14131_c0_seq1_m.31051	ho|comp10145_c0_seq1_m.19254	PF00689	Cation transporting ATPase, C-terminus	g9338.t1	*other*
**cluster104**	2	hv|comp17832_c0_seq1_m.8291	hm|comp12790_c0_seq1_m.23705	-	PF00650	CRAL/TRIO domain	g8780.t1	*other*
**cluster96**	2	hv|comp24042_c1_seq2_m.28281	-	ho|comp10206_c0_seq1_m.19571	PF13906	C-terminus of AA_permease	g10433.t1	*other*
**cluster94**	2	hv|comp24869_c0_seq1_m.32410	-	ho|comp5010_c0_seq1_m.5724	PF01485	IBR domain, a half RING-finger domain	g7258.t1	*other*
**cluster81**	2	hv|comp24495_c0_seq1_m.30505	-	ho|comp11411_c0_seq1_m.25611	PF00225	Kinesin motor domain	g9012.t1	*other*
**cluster84**	2	hv|comp13475_c0_seq1_m.3667	hm|comp8634_c0_seq1_m.10714	-	PF00412	LIM domain	g529.t1	*other*
**cluster82**	2	hv|comp21502_c3_seq1_m.17810	hm|comp13403_c0_seq1_m.26788	-	PF06809	Neural proliferation	-	*other*

						differentiation control-1 protein (NPDC1)		
**cluster100**	2	hv|comp8063_c0_seq1_m.1828	hm|comp6351_c0_seq1_m.6195	-	Q54VU4	Probable serine/threonine-protein kiN/Ase DDB_G0280133	-	*other*
**cluster97**	2	hv|comp18362_c0_seq1_m.9408	-	ho|comp6990_c0_seq1_m.9894	PF03134	TB2/DP1, HVA22 family	g6552.t1	*other*
**cluster78**	2	hv|comp14317_c0_seq2_m.4270	-	ho|comp9276_c1_seq2_m.16313	PF00091	Tubulin/FtsZ family, GTPase domain	-	*other*

Supplementary Table 11. Clusters of orthologs identified by combined transcriptomic and proteomic analysis in the salivary cells and salivary cell secretions of three medicinal leech species.

claster N	number of orthologs	H.verbana id	H.medicinalis id	H.orientalis id	PFAM/ UniProt id	description	gene model id*	category
**cluster138**	2	hv|comp18002_c0_seq1_m.8615	hm|comp11929_c0_seq1_m.20324	-	N/A	Adenosine deaminase	g9375.t1	*Adenosine deaminase*
**cluster112**	2	hv|comp17295_c1_seq1_m.7426	-	ho|comp5324_c1_seq1_m.6297	N/A	Antistasin	g4392.t1	*Antistasin*
**cluster142**	2	hv|comp15474_c0_seq1_m.5206	hm|comp7182_c0_seq1_m.7670	-	PF00188	Cysteine-rich secretory protein family	g4616.t1	*CRISP*
**cluster137**	2	hv|comp20744_c1_seq3_m.15259	hm|comp9470_c1_seq1_m.12719	-	N/A	Cysteine-rich secretory protein family	g7911.t2	*CRISP*
**cluster63**	3	hv|comp12881_c0_seq1_m.3334	hm|comp7049_c0_seq1_m.7418	ho|comp7111_c0_seq1_m.10212	PF00188	Cysteine-rich secretory protein family	g4615.t1	*CRISP*
**cluster7**	6	hv|comp17477_c0_seq1_m.7726 ; hv|comp12226_c1_seq1_m.3024	hm|comp11142_c0_seq3_m.17571 ; hm|comp11142_c0_seq1_m.17569	ho|comp9826_c0_seq2_m.17980 ; ho|comp9826_c0_seq1_m.17975	PF00188	Cysteine-rich secretory protein family	g6998.t1	*CRISP*
**cluster130**	2	hv|comp14827_c0_seq1_m.4651	hm|comp11304_c0_seq1_m.18032	-	N/A	Proteinase inhibitor I13	g6938.t1	*eglin-like*
**cluster73**	3	hv|comp16679_c0_seq1_m.6518	hm|comp13057_c0_seq2_m.25114	ho|comp8497_c0_seq2_m.13852	PF03662	Glycosyl hydrolase family 79, N-terminal domain	g6481.t1	*hyaluronidase*
**cluster119**	2	hv|comp23221_c0_seq3_m.24413	hm|comp12557_c0_seq1_m.22729	-	PF00057	Low-density lipoprotein receptor domain class A	g6099.t1	*Low density lipoprotein receptor A*
**cluster35**	4	hv|comp23931_c0_seq3_m.27754 ; hv|comp23931_c0_seq1_m.27751	hm|comp13369_c0_seq3_m.26639 ; hm|comp13369_c0_seq2_m.26634	-	Q20930	ADAM family mig-17/m12	g13466.t1	*protease M12*
**cluster129**	2	hv|comp8040_c0_seq1_m.1822	hm|comp7967_c0_seq1_m.9253	-	PF01421	Reprolysin (M12B) family zinc metalloprotease	g10940.t1	*protease M12*
**cluster71**	3	hv|comp20559_c0_seq1_m.14759	hm|comp13677_c0_seq3_m.28162	ho|comp9916_c0_seq1_m.18393	PF05649	Peptidase family M13	g8215.t1	*protease M13*
**cluster56**	3	hv|comp23965_c0_seq1_m.27885 ; hv|comp23965_c0_seq2_m.27891	hm|comp13677_c0_seq1_m.28161	-	PF05649	Peptidase family M13	g3765.t1	*protease M13*

**cluster127**	2	hv|comp23789_c1_seq1_m.27025	hm|comp14099_c0_seq2_m.30811	-	PF05649	Peptidase family M13	g9361.t2	*protease M13*
**cluster66**	3	hv|comp13907_c0_seq2_m.3963	hm|comp6586_c0_seq1_m.6565	ho|comp7221_c0_seq1_m.10493	N/A	N/A 1	g4084.t1	*na*
**cluster69**	3	hv|comp20738_c0_seq1_m.15238	hm|comp11293_c0_seq1_m.18002	ho|comp8761_c3_seq1_m.14727	N/A	N/A 2	g11480.t1	*na*
**cluster128**	2	hv|comp14220_c1_seq1_m.4182	hm|comp7863_c0_seq1_m.9045	-	N/A	N/A 20	g12648.t1	*na*
**cluster120**	2	hv|comp12693_c0_seq1_m.3241	hm|comp8279_c0_seq1_m.9970	-	N/A	N/A 23	g2938.t1	*na*
**cluster1**	14	hv|comp15765_c3_seq1_m.5495 ; hv|comp15765_c3_seq5_m.5500	hm|comp7525_c1_seq11_m.8366 ; hm|comp7525_c1_seq5_m.8360 ; hm|comp7525_c1_seq7_m.8362 ; hm|comp7525_c1_seq1_m.8357 ; hm|comp7525_c1_seq10_m.8365 ; hm|comp7525_c1_seq13_m.8369 ; hm|comp7525_c1_seq12_m.8367 ; hm|comp7525_c1_seq9_m.8363	ho|comp7923_c0_seq10_m.12284 ; ho|comp7923_c0_seq1_m.12275 ; ho|comp7923_c0_seq3_m.12279 ; ho|comp7923_c0_seq6_m.12281	N/A	N/A 3	g13709.t1	*na*
**cluster13**	6	hv|comp13832_c0_seq1_m.3900 ; hv|comp17450_c0_seq2_m.7684	hm|comp10776_c1_seq2_m.16395 ; hm|comp10776_c1_seq1_m.16394	ho|comp3137_c0_seq1_m.2876 ; ho|comp9862_c0_seq3_m.18116	N/A	N/A 4	-	*na*
**cluster21**	4	hv|comp15792_c0_seq1_m.5539	hm|comp10900_c0_seq1_m.16751	ho|comp9329_c0_seq2_m.16470 ; ho|comp9329_c0_seq1_m.16468	N/A	N/A 5	g14451.t1	*na*
**cluster34**	4	hv|comp15782_c0_seq2_m.5525 ; hv|comp15782_c0_seq1_m.5524	hm|comp7052_c0_seq2_m.7425	ho|comp8114_c0_seq4_m.12823	N/A	N/A 6	g6722.t2	*na*
**cluster17**	5	hv|comp18790_c0_seq1_m.10278	hm|comp13252_c0_seq3_m.26033 ; hm|comp13252_c0_seq6_m.26037	ho|comp9111_c0_seq3_m.15801 ; ho|comp9111_c0_seq2_m.15800	N/A	N/A 8	g7937.t1	*na*
**cluster136**	2	hv|comp12508_c0_seq1_m.3145	hm|comp7178_c0_seq1_m.7667	-	Q54M70	Aspartyl aminopeptidase	g12816.t1	*other*
**cluster19**	5	hv|comp17482_c0_seq1_m.7738 ; hv|comp14183_c0_seq1_m.4154	hm|comp6954_c0_seq1_m.7249 ; hm|comp8075_c0_seq1_m.9499	ho|comp5578_c0_seq1_m.6781	N/A	NAD:arginine ADP-ribosyltransferase	g11093.t1	*other*

Supplementary Table 12. Unique genes expressed in the salivary cells of *H.medicinalis*.

Gene model id	mRNA reads count	database	id	description
**g7923**	517	N/A	N/A	N/A
**g13748**	301	N/A	N/A	N/A
**g5080**	253	N/A	N/A	N/A
**g11093**	250	Pfam	PF01129	NAD:arginine ADP-ribosyltransferase
**g14451**	239	N/A	N/A	N/A
**g13457**	189	N/A	N/A	N/A
**g7925**	168	N/A	N/A	N/A
**g7926**	158	N/A	N/A	N/A
**g11594**	157	Pfam	PF01421	Reprolysin (M12B) family zinc metalloprotease
**g12753**	152	N/A	N/A	N/A
**g11094**	127	Pfam	PF01129	NAD:arginine ADP-ribosyltransferase
**g7938**	127	N/A	N/A	N/A
**g9139**	114	UniProt	P81492	Hirudin-like
**g3765**	107	Pfam	PF05649	Peptidase family M13
**g13524**	85	Pfam	PF01391	Collagen triple helix repeat (20 copies)
**g7259**	76	Pfam	PF01401	Angiotensin-converting enzyme
**g14544**	67	N/A	N/A	N/A
**g14328**	63	N/A	N/A	N/A
**g7922**	54	N/A	N/A	N/A
**g7924**	49	N/A	N/A	N/A
**g2001**	40	N/A	N/A	N/A
**g11095**	38	Pfam	PF01129	NAD:arginine ADP-ribosyltransferase
**g14319**	36	N/A	N/A	N/A
**g12991**	35	Pfam	PF01431	Peptidase family M13
**g4584**	34	Pfam	PF00474	Sodium:solute symporter family
**g4388**	31	Pfam	PF06079	Apyrase
**g13859**	25	N/A	N/A	N/A

**g8215**	25	N/A	N/A	N/A
**g13384**	18	N/A	N/A	N/A
**g6047**	18	N/A	N/A	N/A
**g6797**	18	N/A	N/A	N/A
**g12518**	13	Pfam	PF05649	Peptidase family M13
**g9138**	12	UniProt	P81492	Hirudin-like
**g13784**	11	N/A	N/A	N/A
**g3477**	11	Pfam	PF00104	Ligand-binding domain of nuclear hormone receptor
**g11255**	10	N/A	N/A	N/A
**g12324**	9	Pfam	PF05649	Peptidase family M13
**g12649**	9	N/A	N/A	N/A
**g11423**	8	N/A	N/A	N/A
**g2612**	8	N/A	N/A	N/A
**g14357**	7	N/A	N/A	N/A
**g13740**	4	N/A	N/A	N/A

Supplementary Table 13. Clusters of orthologs identified by proteomic analysis in salivary cell secretions of three medicinal leech species.

claster N	number of orthologs	H.verbana id	H.medicinalis id	H.orientalis id	PFAM/ UniProt id	description	gene model id*	category
**cluster16**	5	hv|comp22497_c0_seq1_m.21328	hm|comp13725_c0_seq1_m.28437 ; hm|comp13725_c0_seq2_m.28442	ho|comp6895_c0_seq3_m.9687 ; ho|comp6895_c0_seq1_m.9685	PF07703	Alpha-2-macroglobulin family N-terminal region	g12051.t1	*alpha-2-macroglobulin (a2M)*
**cluster38**	4	hv|comp17370_c0_seq1_m.7558 ; hv|comp17370_c0_seq1_m.7557	hm|comp10037_c0_seq1_m.14144	ho|comp11011_c0_seq1_m.23306	PF07678	A-macroglobulin complement component	g7979.t1	*alpha-2-macroglobulin (a2M)*
**cluster134**	2	hv|comp17697_c1_seq1_m.8061	hm|comp10247_c0_seq1_m.14767	-	PF02822	Antistasin family	g4768.t1	*Antistasin*
**cluster3**	8	hv|comp22600_c0_seq1_m.21760 ; hv|comp19288_c0_seq3_m.11469	hm|comp7679_c1_seq1_m.8660 ; hm|comp7679_c1_seq2_m.8661	ho|comp9244_c0_seq1_m.16164 ; ho|comp9244_c0_seq2_m.16167 ; ho|comp9244_c0_seq3_m.16169 ; ho|comp9244_c0_seq4_m.16173	PF02822	Antistasin family	g9922.t1	*Antistasin*
**cluster20**	4	hv|comp14953_c0_seq1_m.4750 ; hv|comp14953_c0_seq2_m.4751	-	ho|comp7056_c0_seq2_m.10074 ; ho|comp7056_c0_seq3_m.10075	N/A	Therostasin	-	*Antistasin*
**cluster116**	2	hv|comp13896_c0_seq1_m.3946	-	ho|comp5337_c0_seq1_m.6319	N/A	Therostasin	-	*Antistasin*
**cluster48**	3	hv|comp5076_c0_seq1_m.1273 ; hv|comp5076_c0_seq2_m.1275	hm|comp6253_c0_seq1_m.6064	-	N/A	WAP Cys-rich	g3476.t1	*Antistasin*
**cluster135**	2	hv|comp20383_c0_seq1_m.14213	hm|comp13086_c0_seq1_m.25222	-	PF13499	EF-hand domain pair	g10003.t1	*Calmodulin*
**cluster11**	6	hv|comp14377_c1_seq6_m.4319 ; hv|comp14377_c1_seq3_m.4317 ; hv|comp14377_c1_seq5_m.4318 ; hv|comp14377_c1_seq7_m.4321	hm|comp7246_c0_seq1_m.7755 ; hm|comp7246_c0_seq2_m.7756	-	PF00194	Eukaryotic-type carbonic anhydrase	g7193.t1	*carbonic anhydrase*
**cluster115**	2	-	hm|comp11142_c0_seq4_m.17572	ho|comp9826_c0_seq8_m.17987	PF00188	Cysteine-rich secretory protein family	-	*CRISP*
**cluster51**	3	hv|comp19536_c0_seq4_m.12015 ; hv|comp19536_c0_seq3_m.12014	hm|comp17910_c0_seq1_m.33641	-	PF00188	Cysteine-rich secretory protein family	g10878.t1	*CRISP*
**cluster42**	3	hv|comp17213_c0_seq1_m.7287	hm|comp9835_c0_seq2_m.13648 ; hm|comp9835_c0_seq1_m.13647	-	PF00188	Cysteine-rich secretory protein family	g7000.t1	*CRISP*
**cluster8**	6	hv|comp15273_c0_seq3_m.5015 ; hv|comp15273_c0_seq1_m.5013 ; hv|comp15273_c0_seq2_m.5014	hm|comp9437_c0_seq1_m.12642 ; hm|comp9437_c0_seq2_m.12643	ho|comp5703_c0_seq1_m.7080	PF00188	Cysteine-rich secretory protein family	g4751.t1	*CRISP*
**cluster75**	3	hv|comp17251_c0_seq1_m.7328	hm|comp8954_c0_seq1_m.11418	ho|comp7707_c0_seq1_m.11648	PF00188	Cysteine-rich secretory protein	g7001.t1	*CRISP*

						family		
**cluster72**	3	hv|comp14619_c0_seq1_m.4504	hm|comp14944_c0_seq1_m.32021	ho|comp10769_c0_seq3_m.22023	PF00754	F5/8 type C domain	g7916.t1	*F5/8 type C domain like*
**cluster58**	3	hv|comp15746_c0_seq2_m.5480 ; hv|comp15746_c0_seq2_m.5479	hm|comp9278_c0_seq2_m.12219	-	P14328	Spore coat protein SP96/lectin	g11933.t1	*F5/8 type C domain like*
**cluster26**	4	hv|comp18511_c2_seq1_m.9705	hm|comp11442_c1_seq2_m.18518 ; hm|comp11442_c1_seq4_m.18519	ho|comp9491_c0_seq1_m.16936	PF00147	Fibrinogen beta and gamma chains, C-terminal globular domain	g14072.t1	*Ficolin*
**cluster122**	2	hv|comp10076_c0_seq1_m.2340	hm|comp5522_c0_seq1_m.4924	-	PF00042	Globin	g3446.t1	*Globin*
**cluster126**	2	hv|comp13296_c0_seq1_m.3546	hm|comp4318_c0_seq1_m.3402	-	PF03662	Glycosyl hydrolase family 79, N-terminal domain	g5922.t1	*hyaluronidase*
**cluster29**	4	hv|comp16550_c0_seq1_m.6367 ; hv|comp16550_c0_seq2_m.6368	hm|comp6781_c0_seq1_m.6916	ho|comp4949_c0_seq1_m.5618	N/A	Glycosyl hydrolase family 79, N-terminal domain	g5922.t1	*hyaluronidase*
**cluster45**	3	hv|comp12214_c0_seq1_m.3016	hm|comp11138_c0_seq3_m.17552 ; hm|comp11138_c0_seq1_m.17550	-	PF14295	PAN domain	g6052.t1	*PAN-domane*
**cluster139**	2	hv|comp32201_c0_seq1_m.38916	hm|comp2891_c0_seq1_m.2042	-	PF08277	PAN-like domain	g976.t1	*PAN-domane*
**cluster61**	3	hv|comp15773_c0_seq1_m.5514	hm|comp8764_c0_seq1_m.10994	ho|comp10480_c0_seq1_m.20646	N/A	Peptidase family M28	g10407.t1	*protease M28*
**cluster143**	2	hv|comp15773_c0_seq1_m.5516	hm|comp8764_c0_seq1_m.10995	-	PF04389	Peptidase family M28	g10407.t1	*protease M28*
**cluster125**	2	hv|comp13890_c0_seq1_m.3940	hm|comp6953_c0_seq1_m.7241	-	PF00652	Ricin-type beta-trefoil lectin domain	g7423.t1	*R-type lectin*
**cluster53**	3	hv|comp17830_c0_seq1_m.8289 ; hv|comp17830_c0_seq2_m.8290	hm|comp9223_c0_seq1_m.12083	-	PF00080	Copper/zinc superoxide dismutase (SODC)	g7707.t1	*superoxide dismutase (SODC)*
**cluster59**	3	hv|comp4502_c0_seq1_m.1154	hm|comp7740_c0_seq1_m.8805	ho|comp5302_c0_seq1_m.6244	PF00335	Tetraspanin family	-	*tetraspanin*
**cluster37**	4	hv|comp21884_c1_seq2_m.18957 ; hv|comp21884_c1_seq1_m.18955	hm|comp11399_c0_seq1_m.18370	ho|comp11210_c0_seq1_m.24350	PF00092	von Willebrand factor type A domain	g6733.t1	*vWFA-like*
**cluster74**	3	hv|comp25097_c0_seq6_m.33517	hm|comp14077_c0_seq1_m.30596	ho|comp11532_c0_seq3_m.26622	PF08742, PF01826	C8 domain, Trypsin Inhibitor like cysteine rich domain	g10240.t1	*vWFD contain*
**cluster2**	9	hv|comp25097_c0_seq6_m.33516 ; hv|comp25097_c0_seq5_m.33514 ; hv|comp25097_c0_seq4_m.33512	hm|comp14077_c0_seq1_m.30595 ; hm|comp14077_c0_seq3_m.30600	ho|comp11532_c0_seq3_m.26624 ; ho|comp11532_c0_seq2_m.26621 ; ho|comp11532_c0_seq1_m.26617 ; ho|comp11532_c0_seq1_m.26618	PF00094	von Willebrand factor type D domain/C8/	g10240.t1	*vWFD contain*
**cluster22**	4	hv|comp25097_c0_seq3_m.33511	hm|comp14077_c0_seq1_m.30597	ho|comp11532_c0_seq3_m.26623 ; ho|comp11532_c0_seq2_m.26620	N/A	Protease ingibitor 15	g10240.t1	*vWFD contain*
**cluster43**	3	hv|comp25097_c0_seq1_m.33507	hm|comp14077_c0_seq4_m.30601	-	N/A	N/A 21	g10240.t1	*vWFD contain*

			; hm|comp14077_c0_seq5_m.30603					
**cluster28**	4	hv|comp20715_c0_seq3_m.15173 ; hv|comp20715_c0_seq4_m.15175	hm|comp11962_c0_seq1_m.20426	ho|comp10725_c0_seq1_m.21795	N/A	N/A 10	g11991.t1	*na*
**cluster36**	4	hv|comp18576_c1_seq2_m.9842 ; hv|comp18576_c1_seq1_m.9840	hm|comp11521_c0_seq1_m.18840	ho|comp10766_c0_seq1_m.22007	N/A	N/A 12	g5191.t1	*na*
**cluster39**	4	hv|comp17827_c0_seq2_m.8281 ; hv|comp17827_c0_seq1_m.8279	hm|comp11521_c0_seq2_m.18842	ho|comp10766_c0_seq2_m.22009	N/A	N/A 13	g5191.t1	*na*
**cluster62**	3	hv|comp12507_c0_seq1_m.3143	hm|comp11444_c0_seq1_m.18524	ho|comp9862_c0_seq2_m.18114	N/A	N/A 15	g7928.t1	*na*
**cluster64**	3	hv|comp12507_c0_seq1_m.3144	hm|comp11444_c0_seq1_m.18525	ho|comp9862_c0_seq2_m.18113	N/A	N/A 16	g7928.t1	*na*
**cluster12**	6	hv|comp20715_c0_seq3_m.15174 ; hv|comp20715_c0_seq1_m.15169 ; hv|comp20715_c0_seq2_m.15171 ; hv|comp20715_c0_seq4_m.15176	hm|comp11962_c0_seq1_m.20427	ho|comp10725_c0_seq1_m.21796	N/A	N/A 7	g11991.t1	*na*
**cluster60**	3	hv|comp17204_c0_seq2_m.7252	hm|comp10250_c0_seq1_m.14781	ho|comp6141_c0_seq2_m.7983	N/A	N/A 14	g1441.t1	*na*
**cluster70**	3	hv|comp21162_c0_seq1_m.16738	hm|comp12147_c2_seq1_m.21111	ho|comp10527_c0_seq1_m.20887	N/A	N/A 17	g14193.t1	*na*
**cluster124**	2	hv|comp18790_c0_seq1_m.10279	hm|comp13252_c0_seq1_m.26031	-	N/A	N/A 19	g7938.t1	*na*
**cluster123**	2	hv|comp18895_c0_seq1_m.10510	hm|comp13132_c0_seq3_m.25451	-	N/A	N/A 24	g2705.t2	*na*
**cluster131**	2	hv|comp17450_c0_seq2_m.7685	hm|comp10776_c1_seq1_m.16393	-	N/A	N/A 25	-	*na*
**cluster144**	2	hv|comp22811_c0_seq2_m.22603	hm|comp4621_c0_seq1_m.3703	-	N/A	N/A 27	g5894.t1	*na*
**cluster113**	2	-	hm|comp9492_c0_seq1_m.12768	ho|comp5332_c0_seq1_m.6310	N/A	N/A 28	-	*na*
**cluster40**	3	hv|comp16746_c0_seq1_m.6606	-	ho|comp9820_c0_seq2_m.17954 ; ho|comp9820_c0_seq1_m.17953	N/A	N/A 29	-	*na*
**cluster57**	3	hv|comp18707_c0_seq1_m.10104 ; hv|comp18707_c0_seq4_m.10107	-	ho|comp7752_c0_seq1_m.11763	N/A	N/A 30	-	*na*
**cluster111**	2	hv|comp16414_c0_seq1_m.6227	-	ho|comp7604_c0_seq3_m.11405	N/A	N/A 31	-	*na*
**cluster24**	4	hv|comp23410_c1_seq1_m.25328	hm|comp7698_c0_seq2_m.8710 ; hm|comp7698_c0_seq1_m.8709	ho|comp9625_c0_seq1_m.17344	N/A	N/A 9	g6936.t1	*na*
**cluster67**	3	hv|comp18771_c0_seq1_m.10245	hm|comp11821_c0_seq1_m.19872	ho|comp7839_c0_seq1_m.12007	PF00171	Aldehyde dehydrogenase family	g3624.t1	*other*
**cluster68**	3	hv|comp18771_c0_seq1_m.10246	hm|comp11821_c0_seq1_m.19870	ho|comp7839_c0_seq1_m.12008	PF00171	Aldehyde dehydrogenase family	g3624.t1	*other*
**cluster133**	2	hv|comp23307_c0_seq2_m.24810	hm|comp13015_c0_seq1_m.24875	-	PF00012	Hsp70 protein	-	*other*
**cluster140**	2	hv|comp15766_c2_seq1_m.5502	hm|comp9614_c0_seq2_m.13067	-	PF00244	14-3-3 protein	g3791.t1	*other*

**cluster46**	3	hv|comp15445_c1_seq1_m.5168	hm|comp11805_c0_seq2_m.19813 ; hm|comp9614_c0_seq3_m.13068	-	PF00244	14-3-3 protein	g3990.t1, g11995.t1	*other*
**cluster6**	6	hv|comp19537_c1_seq1_m.12017 ; hv|comp19537_c1_seq2_m.12019	hm|comp9678_c0_seq1_m.13225	ho|comp8995_c0_seq4_m.15431 ; ho|comp8995_c0_seq2_m.15426 ; ho|comp8995_c0_seq3_m.15429	PF00022	Actin	g4376.t1	*other*
**cluster114**	2	-	hm|comp5751_c0_seq1_m.5242	ho|comp3135_c0_seq1_m.2874	PF00306, PF00006	ATP synthase alpha/beta chain, C terminal domain, ATP synthase alpha/beta family, nucleotide-binding domain	g2850.t1	*other*
**cluster27**	4	hv|comp18585_c1_seq3_m.9865 ; hv|comp18585_c1_seq1_m.9859 ; hv|comp18585_c1_seq2_m.9862	hm|comp12822_c1_seq2_m.23878	-	PF03114	BAR domain	g4525.t1	*other*
**cluster132**	2	hv|comp22110_c2_seq3_m.19788	hm|comp13803_c0_seq3_m.28868	-	PF07716	Basic region leucine zipper	g2990.t1	*other*
**cluster117**	2	-	hm|comp6941_c0_seq1_m.7226	ho|comp7304_c0_seq1_m.10682	PF15511	Centromere kinetochore component CENP-T histone fold	g9617.t1	*other*
**cluster41**	3	-	hm|comp14028_c0_seq2_m.30226	ho|comp10061_c0_seq2_m.18948 ; ho|comp10061_c0_seq3_m.18950	PF00241	Cofilin/tropomyosin-type actin-binding protein	g7441.t1	*other*
**cluster47**	3	hv|comp18349_c1_seq1_m.9390	hm|comp12490_c0_seq3_m.22497 ; hm|comp12490_c0_seq1_m.22494	-	PF00012	Hsp70 protein	g7896.t1	*other*
**cluster121**	2	hv|comp21722_c1_seq1_m.18460	hm|comp13774_c0_seq3_m.28694	-	PF12777, PF12775, PF12780	Microtubule-binding stalk of dynein motor, P-loop containing dynein motor region D3, P-loop containing dynein motor region D4	g433.t1	*other*
**cluster65**	3	hv|comp19136_c0_seq3_m.11117	hm|comp12041_c0_seq1_m.20713	ho|comp5809_c0_seq1_m.7312	PF00071	Ras family	g7577.t1	*other*
**cluster141**	2	hv|comp12418_c0_seq1_m.3084	hm|comp5511_c0_seq1_m.4909	-	PF01020, PF00240	Ribosomal L40e family, Ubiquitin family	g4151.t1	*other*
**cluster118**	2	hv|comp17458_c0_seq2_m.7693	hm|comp6601_c0_seq1_m.6595	-	PF01599, PF00240	Ribosomal protein S27a, Ubiquitin family	-	*other*

Supplementary Results

Accessory proteins

vWFD domain: We identified sequences that encode parts of a larger protein, which is the product of a single gene (g10240.t1) and a large, complex protein that comprises several domains, such as the von Willebrand factor type D domain, C8 domain, and trypsin inhibitor-like Cys-rich domain. The combination of all such domains within a single protein features spondins and mucins. Most secreted mucins are major components of mucus and mucosa and are found in salivary gland secretions, where they perform diverse functions [1].

Calreticulin: This protein family has various functions and is involved in diverse processes. The intracellular functions include chaperon activity, and these proteins play an important role in embryogenesis [2, 3]. A number of studies have shown that calreticulin plays an active role in haemostasis and interacts with numerous coagulation and endothelial factors [4]. In vivo experiments revealed that these proteins exhibit antithrombotic activity [5]. Due to this property, calreticulin has been patented as an anticoagulant. This protein family is often found in omics data for haematophagous species [6, 7]. They may play a modulating role in host haemostasis through binding calcium ions, which act as coagulation cofactors. Leech calreticulin was demonstrated to interact with the complement component C1q and plays an important role in restoring normal synaptic connections in the central nervous system of the leech [8].

Calmodulin: Calmodulin is a conserved multifunctional calcium binding protein containing the EF-hand domain. This protein was shown to activate intracellular eNOS in endothelial cells and to regulate collagen-dependent platelet aggregation [9]. Calmodulin, like calreticulin, is presumed to participate in the removal of free calcium ions.

Thioredoxin-like domain containing protein: Thioredoxin transfers electrons, acts as a chaperone, and, when coupled with glutathione, is a strong antioxidant involved in reactive oxygen species scavenging [10]. This protein has been found to play an important role in the immune response that emerged from peptide antigen–class I MHC interactions [11] and to participate in platelet activation and regulation [12]. Human proteins homologous to thioredoxin (for example, disulfide-isomerase A6) play a role in platelet aggregation induced by agonists, such as convulxin, collagen, and thrombin [13]. Some human extracellular disulfide isomerases are known to mediate platelet aggregation and thrombus formation (ERp57) [14]. They are secreted by platelets on the surface of the membrane and interact with calnexin and calreticulin.

In addition to the listed proteins, we identified globin, EGF-like protein, arginine ADP-ribosyltransferase, aspartyl aminopeptidase, and aldehyde dehydrogenase. The role of these molecules in haemostasis is currently unclear. In both proteomic and transcriptomic data, we identified cytoskeletal proteins, transcription factors, heat shock proteins, and ribosomal proteins.

Supplementary Methods

Determination of the leech's species

Determination of the leech's species was performed by PCR amplification and subsequent sequencing of the gene regions of nuclear and mitochondrial ribosomal RNA [2]. For amplification of the mitochondrial

genes 12S-rRNA we used oligonucleotides 12A and 12B. For amplification of ITS2 nuclear gene we used oligonucleotides IS-A and IS-B. The amplified DNA fragments were sequenced using the same oligonucleotides and compares the sequence contained in the GenBank database: Hirudo medicinalis - AY763166 (IS2) and AY763156 (12S), Hirudo orientalis - AY763170 (IS2) and AY763163 (12S), Hirudo verbana - AY763167 (IS2) and (12S).

12A – 5’ GCCAGCAGCCGCGGTTA
12B – 5’ CCTACTTTGTTACGACTTAT
IS-A – 5’ TGGGTCGATGAAGAGCGCAG
IS-B – 5’ GTAATCCGGTCTGATCTCAG

Sanger sequencing

The sequences of the amplicons were obtained by Sanger dideoxy sequencing method using Big DyeTM Terminator v.3.1 Cycle Sequencing Kit and ABI Prism Genetic Analyzer 3730XL following the manufacturer's instructions (Applied Biosystem, USA).

Genomic DNA extraction from Hirudo medicinalis

Genomic DNA was isolated using standard molecular biology technique with modifications. Pre-sliced leech body (or 15-20 newborn leeches) were placed in a 50 ml tube and 10 ml of SDS-extraction buffer (100 mM NaCl, 10 mM Tris-Cl pH 8, 25 mM EDTA pH 8, 0.5% SDS) and 150 µL of proteinase K (Amresco, USA; 20 mg/ml) were added. The sample was incubated at 37°C for 18 h, inverting the tube three or four times during the first 2 h. Then, 10 ml of phenol was added and mixed by inverting the tube gently for 10 min. After centrifugation at 3500 g for 15 min, the aqueous phase containing the DNA was extracted a second time with 10 ml of phenol:chloroform (50:50). The white aqueous phase was transferred into a new tube and an equal volume of cold (-20°C) 98% ethanol was added. The DNA was precipitated for 20 min at -80°C, and spun at 3500 g for 20 min. The supernatant was discarded and the pellet washed with cold 70% ethanol. The ethanol was removed and the pellet resuspended in 1 ml of TE buffer (10 mM Tris-Cl pH8, 1 mM EDTA pH 8).

Cleaning up genomic DNA

To genomic DNA add 1 volume of 2% CTAB, 100 mM Tris 8.0, 20 mM EDTA, 1.4 M NaCI (preferably at 55-65°C). Mix by inversion and incubate at 65°C for 10 min. Add equal volume of chloroform invert; vortex briefly (up to 30 sec). Spin 5 min in mícrofuge to separate phases. Add to supernatant 1.1 volume of 1% CTAB, 50 mM Tris 8.0,10 mM EDTA (preferably at 55-65°C). Mix well by inversion. A precipitate should be visible. Spin 5 min, 3500 g. Complete dissolve the pellet in 0.5 ml of: 10 mM Tris 8.0, 0.1 mM EDTA, 1 M NaCl by heating 30 min, 65°C. Add 0.6 volume isopropanol. lnvert several times. Spin 8 min in microfuge. The supernatant was removed and the pellet washed with 0.5 ml 70% ethanol. The centrifugation and washing were repeated twice. Finally, DNA pellet was resuspended in 200 µL lowTE and heat 65°C for 30 min.

Draft genome annotation

For performing annotation, three sets of “hints” were formed. A hint is a range into genome sequence likely belongs to a genetic feature such as exon, intron etc. The first set was generated by using H. robusta proteins. They were mapped on assembly scaffolds by using BLAT [15] software(version 34x13) with parameters “-t=dnax -q=prot”. BLAT hits were converted into Augustus [16] software hints by using provided with augustus script “blat2hints.pl”.

Second set was generated by using contigs from transcriptome de novo assembly. It was created by assembly reads from RNA-seq libraries with using Trinity [17] software (version r20131110) with default parameters. Contigs from transcriptome assembly aligned with genome assembly scaffolds by using BLAT software (version 34x13) with default parameters. BLAT hits were converted into augusts software hints by using “blat2hints.pl”

Third sets of hints were generated by using RNA-seq reads. Hints were formed by using standard pipeline similar to described in papers [18, 19] based on BLAT read alignments with genome scaffolds. Sets of hints were combined and provided to Augustus software during annotation. Annotaion was performed by using Augustus (version 3.7.1) with parameters “--genemodel=complete --gff3=on --species=fly”.

Laser microdissection for mRNA-seq

To synthesize cDNA libraries from salivary gland cells of three leech species H. verbana, H. orientalis and H. medicinalis we isolated tissue fragments enriched with this cell type using laser microdissection. Leeches were stretched out on metallic foil by two forceps. Than leeches were allowed to attach this foil and briefly moved into liquid nitrogen. Deeply frozen leeches were detached from metallic foil and anterior part of the body containing salivary gland cell was cut by lancet from the rest of the body. Severed anterior part was attached to aluminum base for preparation of cryo-sections using OCT compound at -20°C. 10 µm sagital sections were made from each leech (the temperature of chamber -20°C, temperature of specimen -24°C) and placed on membrane slides (slides with PEN foil having 2 µm thickness). Slides stored at -70°C until need. For laser microdissection slides were briefly thawed and immediately fixed in 100% ice-cold methanol for 1 minute followed by air drying. Then slides rehydrate in DEPK water for 1 minute, stained in 0.5% methylene blue solution in DEPK water for 1 minute, briefly washed in DEPK water and dehydrate in 100% methanol for 3 minutes followed by air drying. Slides were placed into specimen holder of Leica Laser Microdissection System LMD7000. 0.5 µl tubes with caps filled with 30 µl of RNeasy extraction solution were placed into collector holder. Methylene blue administration allowed us to visualize arias on sections enriched with salivary gland cells by strong blue staining of these cells in contrast to adjacent, poorly stained tissues. These areas were isolated into caps filled with extraction buffer by laser microdissection. As a control we isolated muscles from adjacent areas. In all cases we collected material from 10 sections into one cap. Then cap was removed from the holder, the tube was filled with 170 µl of RNeasy extraction solution, the tube was closed with cap and briefly centrifuged. The tube was stored at -70°C until need.

Real-time PCR

RNA isolation from the LMD samples was carried out with Trizol reagent (Thermo Fisher Scientific) according to the manufacturer's instructions. Total RNA was treated with 2 units DNase I (Thermo Fisher Scientific) in the presence of 20 units ribonuclease inhibitor for 30 min at 37 °C, then phenol-chloroform extraction was carried out, followed by ethanol precipitation. The total amount of RNA was determined using Qubit® 2.0 Fluorometer (Life Technologies) and Quant-iT ™ RNA Assay Kit, 5–100 ng (Thermo Fisher Scientific). The synthesis of the first cDNA strand was performed with using the RevertAid RT Reverse Transcription Kit (Thermo Fisher Scientific). For reverse transcription reactions used 30 ng of total RNA, 100 pmol of primer T20, 20 units of a ribonuclease inhibitor and 200 ea Reverse transcriptase. The reaction mixture was incubated at 42 °C for 60 min and the reaction was terminated by heating at 70 °C for 5 min and further used for the Real-time PCR reaction.

Real-time PCR was performed using CFX96 Touch thermocycler (BioRad) with 20 µl reaction mixture with 0.5 units Taq polymerase (Thermo Fisher Scientific), appropriate buffer and dNTP, 10ng cDNA, 5 pM of each primer, and 0.1x SYBR-Green (Thermo Fisher Scientific). The samples were subjected to the following thermal profile for amplification: 95 °C for 120s , followed by 40 cycles of 94 °C for 15s; 58 °C annealing for 20s; 65 °C extension for 60s + detection.

The calculation of the relative change in the level of expression of the target genes (destabilase, hirudin) was carried out on the basis of the results of quantitative real-time PCR. The amount of cDNA corresponding to the transcripts of the target and reference genes was determined by the difference in the threshold reaction cycle (Ct) for each sample. All assays were performed with three technical replications. Data was processed using the 'delta delta Ct' method. Expression level of destabilase and hirudin was determined by using DebRT1 / DebRT2 and Hir1 / Hir2 primers, respectively. For data normalisation, house-keeping gene (reference gene) tubulin was used with TubHM1 / TubHM2 primers.

primer list:
TubHM1 – 5' CGATGACTCGTTCAACACCT
TubHM2 – 5' CCTTTCCAATGGTATAGTGAC
Hir1 – 5' CTGCGTGTCTCAAGCAATTAC
Hir2 – 5' GGCTTTCAGGGTTCGGTGT
DebRT1 – 5' GAGTTGCGGACCATACCAGA
DebRT2 – 5' GTTCGTCCACCTGTGCAGA

Salivary glands secret sample prepare and digestion

Filter-aided sample preparation (FASP)

Freeze - dried secret leech was resuspended in 500 µL of 50 mM M Tris/HCl, pH 8.0 with 0,1% sodium deoxycholate (DCNa) (Sigma), placed in centrifugal ultrafiltration units with a nominal molecular weight cutoff of 10,000 (Millipore) and centrifuged at 14,000 × g, 20 °C, for 20 min. 400 µL of 50mM Tris/HCl, pH 8.0 with 8 M urea and 0,1% DCNa was added to the filters sample, mixed and centrifuged at 14,000×g, 20°C, for 20 min. The eluates were discarded, 400 µL of 50mM Tris/HCl, pH 8.0 with 0,1% DCNa was added to the filtration unit, and the units were centrifuged again. These two steps were repeated twice. For reducing of protein disulfide bonds 100 µL of 10 mM dithiothreitol (DTT) (BioRad) in 50mM Tris/HCl, pH 8.0 with 0,1% DCNa was added to the filters, samples were incubated at 60°C for 30 min and centrifuged

at 14,000×g, 20°C, for 10 min. For alkylating of cysteine residues 100 µL 55 mM iodoacetamide (BioRad) was added to the filters, samples were incubated at room temperature and in dark for 30 min and centrifuged at 14,000×g, 20°C, for 10 min. Filters were washed twice with 200 µL of 50 mM ammonium bicarbonate with 0,1% DCNa. Proteins were digested in 40 µL of 50 mM ammonium bicarbonate with 0,1% DCNa at 37°C for 16 h, using trypsin (Trypsin Gold, Mass Spectrometry Grade, Promega) at an enzyme to protein ratio of 1:50. The released peptides were collected by centrifugation at 14,000×g for 15 min followed by two washes with 50 µL 0.5 M NaCl. The combined hydrolyzate was desalted using a Discovery DSC-18 Tube (Supelco) according to the manufacturer protocol. Peptides were eluted with 700 µL 75% ACN, 0.1% TFA, dried in a SpeedVac (Labconco) and resuspended in 3% ACN, 0.1% TFA to the final concentration of 5 µg/µL.

Gel-free trypsin digestion using surfactant RapiGest SF (Waters)

Freeze-dried leeches secret was treated with 5 µl of 10% RapiGest SF (Waters) and 1 µl nuclease mix (GE Healthcare) for 30 min at 4 °C, then resuspended in 45 µl of 100 mM ammonium bicarbonate, vortexed and heated at 100 °C for 5 min. After cooling to room temperature insoluble material was removed by centrifugation at 15000 g for 5 min. Supernatant was separated and protein concentration was determined by BCA (BCA Assay Kit, Sigma). Protein cysteine bonds were reduced with 10 mM DTT for 30 min at 60°C and alkylated with 30 mM iodoacetamide in the dark at RT for 30 min. The step with adding DTT was repeated. After alkylation, trypsin (Trypsin Gold, Mass Spectrometry Grade, Promega) was added to supernatant in ratio trypsin : protein equal 1 : 50 and incubated at 37°C for night. For inactivation of trypsin activity and degradation of the acid-labile surfactant stock solution of trifluoroacetic acid (Sigma) (final concentration in sample 0,5%) was added to digest, incubated at 37°C for 45 min and centrifugated at 15000 g for 10 min for removing of surfactant. Hydrolyzate was desalted using a Discovery DSC-18 Tube (Supelco) according to the manufacturer protocol. Peptides were eluted with 700 µL 75% ACN, 0.1% TFA, dried in a SpeedVac (Labconco) and resuspended in 3% ACN, 0.1% TFA to the final concentration of 5 µg/µL.

In-gel digestion of protein sample

Freeze - dried secret leech was resuspended in 500 µL of 50 mM Tris/HCl, pH 6,8, placed in centrifugal ultrafiltration units with a nominal molecular weight cutoff of 3,000 (Millipore) and centrifuged at 14,000 × g, 20°C, for 20 min. This step was repeated twice. Protein concentration in concentrated sample was determined by Bradford (Bradford Protein Assay Kit, BioRad). Then Laemmli buffer was added to concentrated sample (1 : 1), heated for 5 min at 95 ° C and centrifuged at 15,000 g for 10 min. The supernatant was separated and proteins separated by 7.5 % SDS-PAGE. After electrophoresis was stopped the gel was stained with Coomassie G- 250.

For digestion by trypsin, gel slices for each sample were cut into 3 pieces. Each of these pieces was cut into small (1 x 1 mm) cubes and transferred into tube. Gel samples were destained with 50% ACN in 50 mM ammonium bicarbonate at 50 ° C. Protein disulfide bonds were reduced with 10 mM DTT (BioRad) in 100 mM ammonium bicarbonate at 56°C for 30 min and subsequently alkylated with 55 mM IAA in 100 mM ammonium bicarbonate at room temperature for 20 min (in complete darkness). To neutralize unbound IAA 10 mM DTT solution in 100 mM ammonium bicarbonate was added to gel sample again. Upon alkylation the gel samples dehydrated by addition of neat ACN. After removal of the ACN, the samples were subjected to in-gel digestion. Solution of trypsin (20 nanog / µl) (Trypsin Gold, Mass Spectrometry Grade, Promega) in 40 mM ammonium bicarbonate and 10 % ACN was added to the dried gel pieces so that the solution covered the gel pieces, and incubated at first 1 hour on ice, and then 16 hours at 37 °C. The resulting tryptic peptides extracted from the gel successively the following solutions :

5 % formic acid,
50 % ACN and 5 % formic acid,
75 % ACN and 5 % formic acid.
All solutions were prepared on water mQ. Finally the extracted peptides were combined, dried on a vacuum concentrator CentriVap (Labconco) and dissolved in 50 µl of a solution containing 3% ACN and 0.1% TFA. The obtained peptides extract was desalted using a Discovery DSC-18 Tube (Supelco) according to the manufacturer protocol. Peptides were eluted with 700 µL 75% ACN, 0.1% TFA, dried in a SpeedVac (Labconco) and resuspended in 3% ACN, 0.1% TFA to the final concentration of 5 µg/µL.

<u>Instrumentation and analysis</u>

TripleTOF

Analysis was performed on a TripleTOF 5600+ mass-spectrometer with a NanoSpray III ion source (Sciex, USA) coupled to a NanoLC Ultra 2D+ nano-HPLC system (Eksigent, USA). The HPLC system was configured in a trap-elute mode. For a sample loading buffer and buffer A, the mix of 98.9% water, 1% methanol, 0.1% formic acid (v/v) was used. Buffer B was 99.9% acetonitrile, 0.1% formic acid (v/v). Samples were loaded on a trap column Chrom XP C18 3 mm 120 Å 350 mm*0.5 mm (Eksigent, USA) at a flow rate of 3.5 ul/min over 10 min and eluted through the separation column 3C18-CL-120 (3 µm 120 Å) 0.075 mm*150 mm (Eksigent, USA) at a flow rate of 300 nl/min. The gradient was from 5 to 40% of buffer B in 120 min. The column and the precolumn were regenerated between runs by washing with 95% of buffer B for 7 min and equilibrated with 5% of buffer B for 25 min. Between the samples to ensure the absence of carryover both the column and the precolumn were thoroughly washed with a blank injection trap-elute gradient that included five 7 minute 5-95-95-5%B waves followed by 25 min 5%B equilibration.

Mass spectra were acquired in a positive ion mode. Information-dependent mass-spectrometer experiment included 1 survey MS1 scan followed by 50 dependent MS2 scans. MS1 acquisition parameters were as follows: mass range for analysis and subsequent ion selection for MS2 analysis was 300-1250 m/z, signal accumulation time was 250 ms. Ions for MS2 analysis were selected on the basis of intensity with the threshold of 400 cps and the charge state from 2 to 5. MS2 acquisition parameters were as follows: resolution of quadrupole was set to UNIT (0.7 Da), measurement mass range was 200-1800 m/z, optimization of ion beam focus was to obtain maximal sensitivity, signal accumulation time was 50 ms for each parent ion. Collision activated dissociation was performed with nitrogen gas with collision energy ramping from 25 to 55 V within 50 ms signal accumulation time. Analyzed parent ions were sent to dynamic exclusion list for 15 sec in order to get the next MS2 spectra of the same compound around its chromatographic peak apex (minimum peak width throughout the gradient was about 30 s).

For protein identification, .wiff data files were analyzed with ProteinPilot 4.5 revision 1656 (ABSciex) using search algorithm Paragon 4.5.0.0 revision 1654 (Sciex, USA) and a standard set of identification settings to search against . The following parameters were used: alkylation of cysteine - iodoacetamide, trypsin digestion, TripleTOF 5600 equipment, species: none, thorough search with additional statistical FDR analysis, and therefore detected protein threshold equal or more than 0,05. Peptide identifications were processed with default settings by a ProteinPilot software built-in ProGroup algorithm. The protein identification list was obtained with the threshold reliable protein ID unused score calculated by ProteomicS Performance Evaluation Pipeline Software (PSPEP) algorithm for 1% global FDR from fit. Then accidental decoy proteins identification was removed as an all peptides containing nontryptic digestion,

internal cleavages sites, all modifications except alkylated cysteine and nonmodified cysteine was removed too. All proteins covered with remaining peptides formed final proteins list.

Orbitrap

Peptide separations were carried out using an Ultimate 3000 RSLCnano system (Dionex). The sample was loaded into a trap column (Acclaim PepMap, 2 cm × 75 µm inner diameter, C18, 3 µm, 100 A) (Thermo Scientific) at 2 µl/min with 0.1% formic acid in water. After 5 min, the trap column was set on-line with an analytical column (Zorbax 300SB- C18, 15 cm × 75 µm inner diameter, 3,5µm) (Agilent). Peptide elution was performed by applying a mixture of solvents A and B. Solvent A was HPLC grade water with 0.1% (v/v) formic acid, and solvent B was 80%(v/v) HPLC grade acetonitrile/water with 0.1% (v/v) formic acid. Separations were performed by applying a linear gradient from 5% to 60% solvent B at 400 nl/min over 85 min followed by a washing step (10 min at 99% solvent B) and an equilibration step (25min at 5% solvent B).

MS analyses were performed using a Q-Exactive HF mass spectrometer (Thermo Scientific, Germany). A nanospray Flex ion source with 1800 V ionization voltage and 200°C capillary temperature were used. MS data were acquired in a data-dependent strategy selecting the fragmentation events based on the precursor abundance in the survey scan. MS survey scans were acquired at a resolution of 70000, scan range 400-1200 m/z, target AGC values of 1 × 106, maximum injection time 50 ms. Fragmentation was performed for 25 most abundant ions with a normalized collision energy of 35, dynamic exclusion 10 s. MS/MS scans were acquired with resolution of 17500, target AGC values of 1 × 105, maximum injection time 100 ms, isolation window 2,0 m/z. Obtained mass-spectra in thermo.raw format was converted in non-calibrated peaklists (.mgf extension) with MSconvert program from open source ProteoWizard program bundle. This peaklists were analyzed with ProteinPilot 4.5 revision 1656 (ABSciex) as was mentioned above.

Maxis **(**protein – gel, protein – FASP-DCA-Urea, protein- RapiGest):

Analysis was performed on Maxis 3G mass-spectrometer with HDC-cell upgrade with Online NanoElectrospray ion source (Bruker Daltonics GmbH, Germany) coupled with Dionex Ultimate 3000 (ThermoScientific, USA) HPLC system which was configured in trap-elute mode. For sample loading buffer and buffer A, the mix of 98.9% water, , 1% methanol, 0.1% formic acid (v/v) was used. Buffer B was 99.9% acetonitrile, 0.1% formic acid (v/v). Sample equivalents of 3 micrograms of protein was loaded on a trap column Zorbax 300SB-C18 (3,5 µm, 300 Å) 5mm length (Agilent technologies, USA) at flow rate 20 µl/min of loading buffer over 10 minutes and eluted through the separation column Zorbax 300SB-C18(3,5 µm, 300 Å) 150*0.075mm (Agilent technologies, USA) at flow rate 300 nl/min at room temperature (22°C). The gradient was start from 100% of buffer A during 9 minutes, then from 100% of buffer A to 5% of buffer B in 1 minutes, then from 5% to 50% of buffer B in 40 minutes and finally from 50% to 99% in 5 minutes (for gel pieces).

OR The gradient was start from 100% of buffer A during 9 minutes, then from 100% of buffer A to 5% of buffer B in 1 minutes, then from 5% to 30% of buffer B in 140 minutes and finally from 30% to 99% in 5 minutes (for total proteome FASP-DCA-Urea, RapiGest). The column and precolumn were regenerated between runs by washing with 99% of buffer B for 10 minutes and equilibrated with 100% of buffer A for 20 minutes. Between the different samples to ensure the absence of carryover both the column and the precolumn were thoroughly washed with two blank injection: first was made with trap-elute gradient that included 12 10 minute 0-5-99-0%B waves followed by 25 min 100%A equilibration and the second was

blank injection with standard separation gradient. Between the different pieces of the same gel only blank injection with standard separation gradient was performed.

Ion source parameters were as follows: drying gas (nitrogen) flow rate - 4 l/min, heater temperature - 170°C, ionization voltage - 1700V, positive ion mode. Information-dependent mass-spectrometer experiment included 1 survey MS1 scan followed by 20 dependent MS2 scans with frequency of ToF spectra acquisition 4 Hz, total cycle time 2.2 sec. MS1 acquisition parameters were as follows: mass range for analysis and subsequent ion selection for MS2 analysis was 200-1500 m/z. Ions for MS2 analysis were selected on the basis of intensity with the threshold of 1000 cps and the charge state from 2 to 5. MS2 acquisition parameters were as follows: measurement mass range was 200-1500 m/z. Collision activated dissociation was performed with nitrogen gas with collision energy ramping from 25 to 55 V depending on m/z of precursor ion. Analyzed parent ions were sent to dynamic exclusion list for 0.25 min in order to get the next MS2 spectra of the same compound around its chromatographic peak apex (minimum peak width throughout the gradient was about 30 s). Raw spectra with the use of program Compass DataAnalysis (Bruker Daltonics GmbH, Germany) were converted in non-calibrated peaklists (.mgf extension). This peaklists were analyzed with ProteinPilot 4.5 revision 1656 (ABSciex) as was mentioned above.

Supplementary References

1. Lang T, Hansson GC, Samuelsson T. Gel-forming mucins appeared early in metazoan evolution. Proc Natl Acad Sci. 2007;104:16209–14. doi:10.1073/pnas.0705984104.

2. Park BJ, Lee DG, Yu JR, Jung SK, Choi K, Lee J, et al. Calreticulin, a calcium-binding molecular chaperone, is required for stress response and fertility in Caenorhabditis elegans. Mol Biol Cell. 2001;12:2835–45.

3. Rauch F, Prud'homme J, Arabian A, Dedhar S, St-Arnaud R. Heart, Brain, and Body Wall Defects in Mice Lacking Calreticulin. Exp Cell Res. 2000;256:105–11. doi:10.1006/excr.2000.4818.

4. Kuwabara K, Pinsky DJ, Schmidt AM, Benedict C, Brett J, Ogawa S, et al. Calreticulin, an antithrombotic agent which binds to vitamin K-dependent coagulation factors, stimulates endothelial nitric oxide production, and limits thrombosis in canine coronary arteries. J Biol Chem. 1995;270:8179–87.

5. Dai E, Stewart M, Ritchie B, Mesaeli N, Raha S, Kolodziejczyk D, et al. Calreticulin, a potential vascular regulatory protein, reduces intimal hyperplasia after arterial injury. Arterioscler Thromb Vasc Biol. 1997;17:2359–68.

6. Kazimírová M, Štibrániová I. Tick salivary compounds: their role in modulation of host defences and pathogen transmission. Front Cell Infect Microbiol. 2013;3:43. doi:10.3389/fcimb.2013.00043.

7. Jaworski DC, Simmen FA, Lamoreaux W, Coons LB, Muller MT, Needham GR. A secreted calreticulin protein in ixodid tick (Amblyomma americanum) saliva. J Insect Physiol. 1995;41:369–75. doi:10.1016/0022-1910(94)00107-R.

8. Lefebvre C, Bocquet-Garcon A, Vizioli J, Vancamp C, Drago F, Franck J, et al. Calreticulin contributes to C1q-dependent recruitment of microglia in the leech Hirudo medicinalis following a CNS injury. Med Sci Monit. 2014;20:644–53. doi:10.12659/MSM.890091.

9. Gardiner EE, Arthur JF, Berndt MC, Andrews RK. Role of calmodulin in platelet receptor function. Curr Med Chem Cardiovasc Hematol Agents. 2005;3:283–7. http://www.ncbi.nlm.nih.gov/pubmed/16250859. Accessed 19 Apr 2018.

10. Espinosa-Diez C, Miguel V, Mennerich D, Kietzmann T, Sánchez-Pérez P, Cadenas S, et al. Antioxidant responses and cellular adjustments to oxidative stress. Redox Biol. 2015;6:183–97. doi:10.1016/j.redox.2015.07.008.

11. Dick TB. Assembly of MHC class I peptide complexes from the perspective of disulfide bond formation. Cell Mol Life Sci. 2004;61:547–56. doi:10.1007/s00018-003-3271-9.

12. Mor-Cohen R. Disulfide Bonds as Regulators of Integrin Function in Thrombosis and Hemostasis. Antioxid Redox Signal. 2016;24:16–31. doi:10.1089/ars.2014.6149.

13. Jordan PA, Stevens JM, Hubbard GP, Barrett NE, Sage T, Authi KS, et al. A role for the thiol isomerase protein ERP5 in platelet function. Blood. 2005;105:1500–7. doi:10.1182/blood-2004-02-0608.

14. Wu Y, Ahmad SS, Zhou J, Wang L, Cully MP, Essex DW. The disulfide isomerase ERp57 mediates platelet aggregation, hemostasis, and thrombosis. Blood. 2012;119:1737–46. doi:10.1182/blood-2011-06-360685.

15. Kent WJ. BLAT--the BLAST-like alignment tool. Genome Res. 2002;12:656–64. doi:10.1101/gr.229202.

16. Stanke M, Morgenstern B. AUGUSTUS: a web server for gene prediction in eukaryotes that allows user-defined constraints. Nucleic Acids Res. 2005;33 Web Server issue:W465-7. doi:10.1093/nar/gki458.

17. Grabherr MG, Haas BJ, Yassour M, Levin JZ, Thompson DA, Amit I, et al. Full-length transcriptome assembly from RNA-Seq data without a reference genome. Nat Biotechnol. 2011;29:644–52. doi:10.1038/nbt.1883.

18. Stanke M, Diekhans M, Baertsch R, Haussler D. Using native and syntenically mapped cDNA alignments to improve de novo gene finding. Bioinformatics. 2008;24:637–44. doi:10.1093/bioinformatics/btn013.

19. Stanke M, Tzvetkova A, Morgenstern B. AUGUSTUS at EGASP: using EST, protein and genomic alignments for improved gene prediction in the human genome. Genome Biol. 2006;7 Suppl 1 Suppl 1:S11.1-8. doi:10.1186/gb-2006-7-s1-s11.
