# Supplementary figures and images for: Draft genome sequences of Hirudo medicinalis and salivary transcriptome of three closely related medicinal leeches
Source: BMC Genomics. 2020 Apr 29;21:331. doi: 10.1186/s12864-020-6748-0 (PMC7191736; doi:10.1186/s12864-020-6748-0)

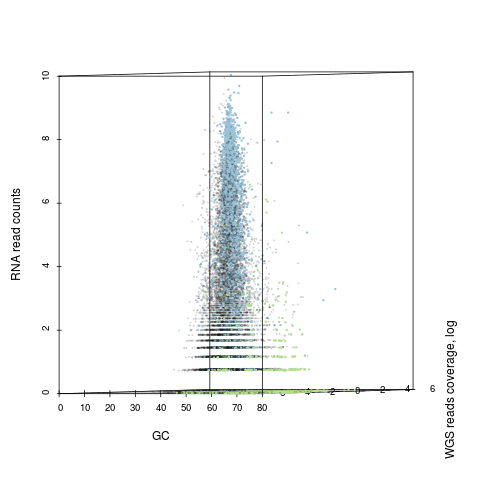

Supplement: Supplementary file 2 — Additional file 2. Supplementary Data 2. The 3D plot showing the contig distribution in coordinates of GC content, read coverage (Proton and Illumina), and host cDNA read coverage. [file 12864_2020_6748_MOESM2_ESM.gif]
